# Inhibition of MEKK3 Prevents Cerebral Cavernous Malformation Progression Via Restoring Endothelial Integrity and Restricting Microglial Activation

**DOI:** 10.1002/advs.78132

**Published:** 2026-10-06

**Authors:** Weiwei Zheng, Suyun Yu, Liwenyu Chen, Jing Ma, Peiliang Shen, Chang Yu, Xiaoqing Xin, Qiuhong Shen, Jing Chen, Song Ke, Hao Liu, Xiaobing Jiang, Ruogu Qi, Juming Yan, Jia Li, Jaesung P. Choi, Yin Lu, Yang Zhao

**Affiliations:** ^1^ Jiangsu Key Laboratory for Pharmacology and Safety Research of Chinese Materia Medica Nanjing University of Chinese Medicine Nanjing China; ^2^ Department of Biochemistry and Molecular Biology School of Medicine Nanjing University of Chinese Medicine Nanjing China; ^3^ Matwings Technology Company Shanghai China; ^4^ State Key Laboratory of Oncology in South China Collaborative Innovation Center for Cancer Medicine Department of Neurosurgery Sun Yat‐sen University Cancer Center Guangzhou China; ^5^ Jiangsu Key Laboratory of Immunity and Metabolism Department of Pathogenic Biology and Immunology National Experimental Demonstration Center for Basic Medicine Education Xuzhou Laboratory of Infection and Immunity Xuzhou Medical University Xuzhou China; ^6^ Curtin Medical Research Institute and Curtin Medical School Curtin University Bentley WA Australia; ^7^ School of Biomedical Sciences University of New South Wales Sydney NSW Australia; ^8^ State Key Laboratory of Mechanism and Quality of Chinese Medicine Macau University of Science and Technology Taipa Macau SAR China

**Keywords:** cerebral cavernous malformation, DPDH, endothelial cells, fibrinogen, MEKK3, microglial cells

## Abstract

Cerebral cavernous malformations (CCMs) are characterized by aberrant MEKK3 activation, yet how MEKK3 governs lesion maturation and whether it represents a viable therapeutic target remain unclear. We demonstrate that endothelial MEKK3 initiates lesion formation, whereas microglial MEKK3 exacerbates late‐stage progression. Mechanistically, MEKK3 activation in endothelial cells disrupts junctional integrity and promotes fibrinogen leakage; fibrinogen‐recruited microglia subsequently amplify vascular injury via MEKK3–NF‐κB–dependent uptake of damaged endothelial debris. Through AI‐assisted drug design and structural optimization, we identified (R)‐1,6‐dimethyl‐11‐phenyl‐1,10‐dihydro‐2H‐furo[2′,3′:1,2]phenanthrol[3,4‐d]imidazole (DPDH) as a potent MEKK3 inhibitor. DPDH suppresses MEKK3‐KLF2/4 signaling in endothelial cells to restore barrier integrity and inhibits MEKK3–NF‐κB in microglia to restrain their activation and uptake of damaged endothelial cells. Collectively, our findings establish that MEKK3 signaling in both endothelial cells and microglia drives CCM progression, and position DPDH as a promising therapeutic candidate for CCM treatment.

## Graphical abstract

In this study, we demonstrate that aberrant MEKK3 signaling in endothelial cells and microglia drives the progression of cerebral cavernous malformations (CCMs). Furthermore, we identify DPDH as a potent small‐molecule MEKK3 inhibitor with substantial therapeutic potential for CCM treatment.

## 1 Introduction

Cerebral cavernous malformations (CCMs) are common vascular lesions featured with aberrantly dilated and leaky capillaries that arise predominantly in the central nervous system, with a prevalence of approximately 0.5% in the human population [1]. CCMs are comprised of clusters of enlarged endothelial channels in low‐blood flow venous capillaries accompanied by poor coverage of pericytes and smooth muscle cells (SMCs) as well as disrupted adherens junctions (AJs) and tight junctions (TJs), leading to elevated permeability, intracranial hemorrhage, and subsequent neurologic deficits [2, 3]. Currently, microsurgical resection serves as the preferred treatment option for symptomatic CCMs [4, 5]. Nevertheless, surgical excision poses an increased risk of brain injury, and not all cavernomas can be safely and completely removed [6]. As such, effective and efficient pharmacological interventions for the management of CCMs are urgently required.

It has been widely held that inherited germline and somatic loss‐of‐function mutations in one of the three known *CCM* genes, namely *KRIT1* (Krev1 interaction trapped gene 1, *CCM1*), *CCM2* (malcavernin), *PDCD10* (programmed cell death protein 10, *CCM3*), are closely correlated to the development CCMs [7, 8]. Indeed, CCMs have been proven to be endothelial‐autonomous disorders due to that endothelial‐specific inactivation of murine *KRIT1*, *CCM2*, or *PDCD10* is prone to contribute to prominent vascular lesions in brain and retina that are similar to those observed in patients with CCMs [9, 10]. Mechanistically, the three *CCM* genes are capable of encoding non‐homologous cytoplasmic proteins, which further generate an active signaling complex to function as an endogenous inhibitor of the MEKK3 [also called MAP3K3 (mitogen‐activated protein kinase kinase kinase 3)] kinase cascade. More specifically, endothelium‐specific conditional knockout of any of the three *CCM* genes result in activation of the MEKK3 kinase cascade as evidenced by the augmented expression levels of an array of downstream targets including Krüppel‐like factor (KLF) 2/4 and phospho‐myosin light chain (pMLC), which may subsequently disrupt endothelial homeostasis [9, 11, 12]. Notably, these critical signaling molecules in endothelial cells (ECs) are of great importance in driving the formation of CCM lesions characterized by isolated single caverns that range in size (called Stage 1 lesions) [13]. It has been shown that genetic ablation of endothelial *MEKK3*‐*KLF* signaling in mice is able to limit the formation of CCM lesions.

It has been increasingly recognized that CCM lesions also acquire the multi‐cavernous stage (Stage 2 lesions), which are characterized by deposition of hemosiderin and infiltration of various types of immune cells in both mouse and human CCM lesions, but the mechanistic linkage remains a long‐standing mystery [7]. Recent studies in the CCM animal models have proposed an EC‐non‐autonomous mechanism underlying the formation of CCM lesions in the central nervous system [14, 15]. For instance, it has been documented that ECs derived from the CCM mouse brains display enhanced expression levels of multiple inflammation‐associated genes that encode corresponding proinflammatory chemokines and cytokines, which paves the way for inducing the recruitment of neutrophils [16]. Additionally, neutrophil extracellular traps (NETs), mainly produced by neutrophils, are prone to deposit in the cerebral cavernomas of CCM mice, and they can be degraded following the treatment of DNase I to further enhance the vascular integrity [16]. All of these imply that the crosstalk between ECs and immune cells may be essential for the progression of CCMs. In fact, histological analysis of CCM lesions in chronic mouse models reveals a striking accumulation of ionized calcium‐binding adapter molecule 1 (IBA‐1)^+^ microglial cells in the proximal regions of CCM lesions. More interestingly, the IBA‐1^+^ microglial cells located around the thrombus in CCM lesions are found to be hypertrophied and ameboid in terms of morphology [17]. It has been well accepted that microglial cells tend to respond to brain injury, but it is ambiguous if they are involved in regulating the integrity of blood–brain barrier (BBB) in CCM lesions, which may be fundamental for exacerbation of neuroinflammatory responses in CCM disease. Of note, reactive microglial cells have been shown to result in leaky BBB in various types of diseased brains by virtue of downregulating a series of TJs‐associated proteins including Claudin‐5, occludin, and zonula occludens‐1 (ZO‐1) [18, 19]. Moreover, as characterized in neuronal diseases and brain trauma, phagocytic microglial cells are also inclined to engulf ECs as well as other important components in the neurovascular unit (NVU) [20, 21]. Nonetheless, the interactions between reactive microglial cells and ECs in influencing the progression of CCMs still require definition. Of interest, it has been reported that MEKK3 plays a pivotal role in promoting the production of lipopolysaccharide (LPS)‐induced interleukin‐6 (IL‐6) and granulocyte–macrophage colony‐stimulating factor (G‐CSF) in the macrophages [22], suggesting MEKK3 may participate in the activation of microglial cells in CCM lesions. To this end, manipulation of MEKK3 in both ECs and microglial cells is likely to emerge as a promising therapeutic approach for fighting against the development of CCMs.

In the present study, we reveal that the abnormally structural and functional blood vessels in the CCM lesions facilitate the leakage of fibrinogen into brain parenchyma in the endothelial‐specific KRIT1 knockout (*iECre;Krit1^fl/fl^* termed *Krit1^iECKO^*) mice. The fibrinogens around the CCM lesions contribute to the recruitment of microglial cells. Subsequently, the activated microglial cells tend to engulf ECs, which further exacerbates the damage of endothelial barrier. Mechanistically, we unveil that MEKK3 exerts a prominent effect on the activation of microglial cells, and its ablation impedes the disturbed endothelium by microglial cells. All of these data indicate that pharmacological suppression of the MEKK3 signaling cascade may provide an effective and efficient therapeutic route for treating CCM disease. Taking advantage of artificial intelligence (AI)‐assisted drug design methodology in combination with structural modification strategy, we obtain (R)‐1,6‐dimethyl‐11‐phenyl‐1,10‐dihydro‐2H‐furo[2′,3′:1,2]phenanthrol[3,4‐d]imidazole (DPDH), a small molecule compound that robustly represses the enzyme activity of MEKK3. On one hand, DPDH is able to target the MEKK3‐KLF2/4 signaling axis in ECs to stabilize the endothelial junctions in CCM lesions. On the other hand, it can also blunt the MEKK3‐NF‐κB signaling cascade in the microglial cells to prohibit the activation of microglial cells, thereby reducing the uptake of EC debris. In conclusion, our study demonstrates that DPDH, as a potent small‐molecule inhibitor of MEKK3, holds great therapeutic potential for treating CCM disease.

## 2 Results

### 2.1 Heterozygous Deletion of Endothelial MEKK3 Prevents the Formation of CCM Lesions Provoked by Induced Knockout of *Krit1* Gene in ECs

Since it has been highly appreciated that neonatal mice with the same induced endothelial‐specific deletions of CCM genes are able to exhibit dramatically different CCM lesion burdens when they are raised in different animal facilities [23], we first determined the temporal course of lesion formation in the *Krit1^iECKO^* mice in the SPF facilities at the Nanjing University of Chinese Medicine, China. Similarly, the deletion of *Krit1* gene was triggered upon the injection of 4‐HT at P1. Vascular malformations were clearly observed at P7 as evidenced by hemorrhagic and dilated venules. Visual appearance of CCM lesions in the hindbrain of *Krit1^iECKO^* mice at P7, P11 and P19 demonstrated that CCM lesion burdens developed over time, as reflected by the increased and expanded blood‐filled caverns in the hindbrain (Figure S1a). This was substantiated by the immunofluorescence staining of CD31 at different time points, showing that the size of CCM lesions enlarged in a time‐dependent manner (Figure S1b). Given that it has been widely held that knockout of CCM genes in ECs results in CCM formation via activating the MEKK3 kinase cascade and elevating the expression levels of downstream targets including KLF2/4 and ADAMTS4/5 [9, 14], we further investigated whether inhibition of MEKK3 kinase cascade could retard the development of CCM lesions at the new animal facilities. As anticipated, it was demonstrated that heterozygous deletion of endothelial MEKK3 conferred to significant reductions in the number and size of CCM lesions that were provoked by induced knockout of *Krit1* gene in ECs (Figure 1a). Consistently, in the retinas of *Krit1^iECKO^* mice, isolectin B4 (IB4) staining revealed that venous malformations developed at the periphery of vascular plexus. However, loss of one allele of MEKK3 gene in ECs was able to effectively retard the lesion formation of retinas in the *Krit1^iECKO^* mice (Figure 1a,e). Of note, CCM lesions are characterized by disorganized endothelium as well as disrupted interactions between ECs and pericytes. To this end, scanning electron microscope (SEM) was employed to visualize the structure of endothelium. It was shown that, in comparison to the *Krit1^fl/fl^* mice, the *Krit1^iECKO^* mice exhibited disorganized endothelium with damaged endothelial junctions. Nevertheless, MEKK3 haploinsufficiency in ECs yielded enhanced vascular integrity with strengthened EC junctions and flatten morphology, indicative of normalized endothelium (Figure 1b).

**FIGURE 1:**
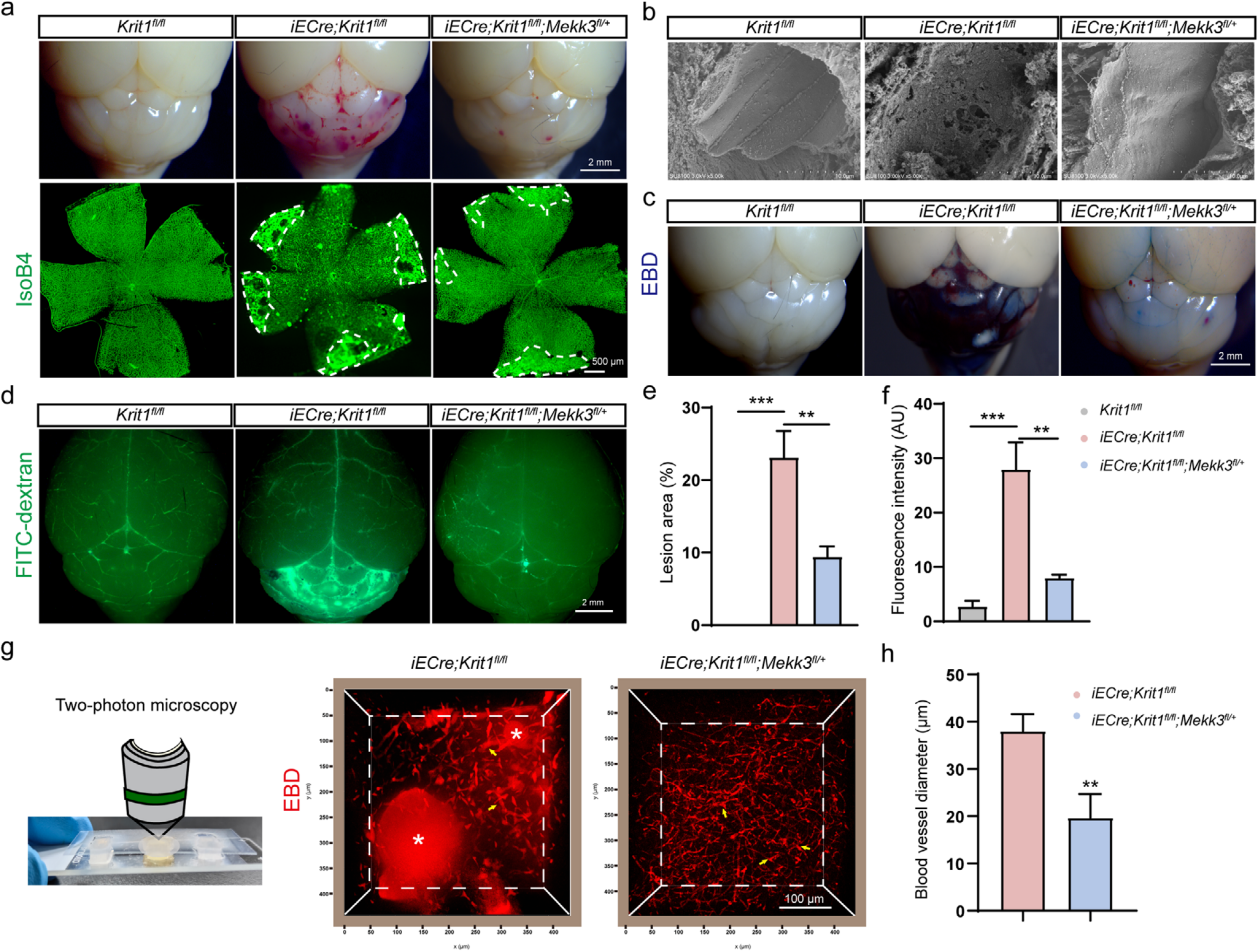
Heterozygous deletion of endothelial MEKK3 prevents the formation of CCM lesions provoked by induced knockout of *Krit1* gene in ECs. a. Top panel: visual detection of CCM lesions in the hindbrains of P19 *Krit1^fl/fl^*, *Krit1^iECKO^*, and *iECre;Krit1^fl/fl^;Mekk3^fl/+^* mice, n = 7–10. Scale bar: 2 mm. Bottom panel: Representative images of whole‐mount immunofluorescence staining for retinas isolated from the *Krit1^fl/fl^*, *Krit1^iECKO^*, and *iECre;Krit1^fl/fl^;Mekk3^fl/+^* mice stained with IB4, n = 4–5. Scale bar: 500 µm. b. Representative SEM images of the blood vessels in the hindbrains from *Krit1^fl/fl^*, *Krit1^iECKO^*, and *iECre;Krit1^fl/fl^;Mekk3^fl/+^* mice, n = 3. c. Visual detection of CCM lesions in the hindbrains of P19 *Krit1^fl/fl^*, *Krit1^iECKO^*, and *iECre;Krit1^fl/fl^;Mekk3^fl/+^* mice following the administration of EBD, n = 6–10. Scale bar: 2 mm. d. Representative fluorescence images of brains harvested from the *Krit1^fl/fl^*, *Krit1^iECKO^* and *iECre;Krit1^fl/fl^;Mekk3^fl/+^* mice following the injection of FITC‐dextran, n = 3–7. Scale Bar: 2 mm. e. Quantification for the percentage of lesion area (refer to a) in the retina was given as the percentage of the marked condensed vascular plexus at the leading edge against the total area of the retinal blood vessels. f. Quantifications for the fluorescence intensity of FITC‐dextran (refer to d) in the hindbrains of *Krit1^fl/fl^*, *Krit1^iECKO^* and *iECre;Krit1^fl/fl^;Mekk3^fl/+^* mice. g. Representative two‐photon microscopy images of vascular malformations in the hindbrains from *Krit1^iECKO^* and *iECre;Krit1^fl/fl^;Mekk3^fl/+^* mice. Arrows indicate caverns and asterisks indicate lesions. Scale bar: 100 µm. h. Quantifications of vein diameters in the hindbrains (refer to g). All data are expressed as mean ± SD. ^**^ *p* <0.01, ^***^ *p* <0.001.

Furthermore, it has been accepted that CCM lesions tend to be formed by enlarged and irregular blood vessels that frequently contribute to cerebral hemorrhage. Therefore, we further evaluated the permeability of brain blood vessels to demonstrate the functions of BBB using a variety of assays. Firstly, the leakage of EBD was employed to determine the vascular permeability in the hindbrains. Intriguingly, visual inspection unveiled that the leakage of EBD in the hindbrains of *Krit1^iECKO^* mice was significantly increased compared to that of *Krit1^fl/fl^* mice. Nonetheless, *iECre;Krit1^fl/fl^;Mekk3^fl/+^* mice displayed prominent reduction in the EBD leakage as compared to the *Krit1^iECKO^* mice (Figure 1c). In agreement with this finding, the amount of FITC‐dextran that leaked from blood vessels into brain parenchyma in the *Krit1^iECKO^* mice was robustly augmented than that in the *Krit1^fl/fl^* mice, which could be rescued following the heterozygous deletion of endothelial MEKK3 (Figure 1d,f). Additionally, to further characterize the architecture and function of blood vessels in the hindbrains, two‐photon microscopy was utilized accordingly. After the injection of EBD, *iECre;Krit1^fl/fl^;Mekk3^fl/+^* mice presented protruding blood‐filled (EBD‐perfused) balloons (caverns) compared to the *Krit1^iECKO^* mice, in which the microvascular network was disrupted with the number and size of caverns being remarkably increased (Figure 1g). More precisely, the lesions in the *Krit1^iECKO^* mice exhibited enlarged veins with a vein diameter approximately two times higher than that of *iECre;Krit1^fl/fl^;Mekk3^fl/+^* mice (Figure 1h; Movies 1 and 2). Collectively, these data highlight that inhibition of MEKK3 signaling in the endothelium hinders the formation of CCM lesions.

### 2.2 DPDH Inhibits the MEKK3 Kinase Activity via Directly Generating Interactions with the Asp44 Residue of MEKK3

Considering that MEKK3 acts as a preponderant therapeutic target for limiting the development of CCM lesions, we intended to identify the potential small‐molecule compounds that exert striking effects on inhibiting the MEKK3 kinase activity. As such, AI‐assisted drug design was employed to design 253 brand‐new small‐molecule compounds that potentially have strong inhibitory effects on MEKK3 kinase activity on the basis of the predicted compound‐protein affinity (Figure 2a and Table S2). Next, the small‐molecule compound with the highest predicted inhibitory effect on MEKK3 kinase activity was selected as a template, whose chemical structure was compared to that of 332 small‐molecule compounds from the traditional Chinese medicine library using the UNITY 2D fingerprint similarity comparison. Among them, the top 10 compounds (No. 63, 180, 57, 9, 103, 324, 50, 223, 185, 99) were selected according to the ranking of similarity score for further analysis (Figure 2b,c). It has been documented that MEKK3 leads to the activation of the extracellular signal‐regulated kinase 5 (ERK5) signaling cascade via phosphorylating and activating the MAPK kinase MEK5 [24]. Thus, in order to further determine the phosphorylation of MEK5 to reflect the MEKK3 kinase activity following the treatment of the 10 screened compounds, we carried out the in vitro kinase assay by virtue of the purified FLAG‐MEKK3 and GST‐MEK5. Notably, the addition of ATP (1 mM) was able to significantly boost the phosphorylation of MEK5, whereas treatment with 10 µM of Dihydrotanshinone I (DHT) was found to possess the strongest capability to reverse the elevated phosphorylation level of MEK5, indicating that DHT could effectively suppress the kinase activity of MEKK3 (Figure 2d). To obtain more potent compounds to inhibit MEKK3 activity, we further modified the chemical structure of DHT to synthesize the following three new compounds. (i):1,6‐dimethylphenanthro[1,2‐b]furan‐10,11‐dione (DPFD); (ii): (R)‐4,9‐dimethyl‐8,9‐dihydrofuro[3,2‐α]naphtho[2,1‐c]phenazine (DDNP); (iii): (R)‐1,6‐dimethyl‐11‐phenyl‐1,10‐dihydro‐2H‐furo[2′,3′:1,2]phenanthro[3,4‐d]imidazole (DPDH) (Figures 2e and S2–S8). To our surprise, the SPR analysis elucidated that DPDH displayed the highest binding affinity to MEKK3 with a KD value of 21.4 µmol/L, surpassing DHT, DPFD, and DDNP (Figure 2f). Consistently, molecular docking data unveiled that DPDH harbored the lowest binding energy (−7.998) to MEKK3 among the four compounds (Figure 2g). Through analyzing the chemical structures of the four compounds, we postulated that DPDH exhibiting the strong affinity to MEKK3 might be ascribed to its specific NH group. More interestingly, it was pinpointed that 10 µM of DPDH exhibited stronger inhibition on MEKK3 kinase activity compared to DHT as validated by the p‐MEK5 level in enzyme activity assay, implying its superior effectiveness over DHT. Further examination found that even 2 µM DPDH presented the inhibitory effect on MEKK3 kinase activity (Figure 2h,i).

**FIGURE 2:**
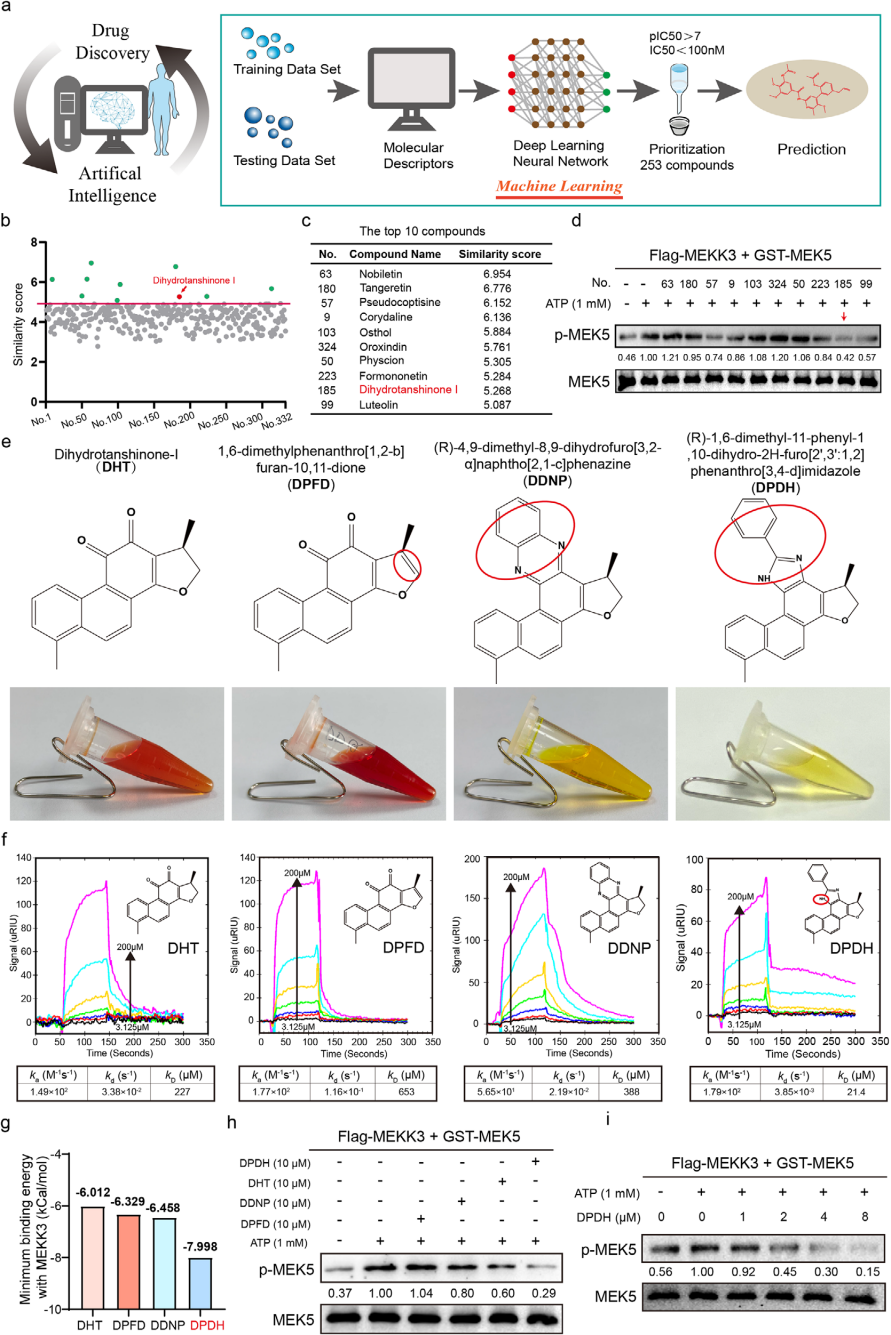
DPDH serves as a potent small‐molecule compound that can bind to MEKK3 to antagonize its kinase activity. a. Schematic diagram of the screening process for MEKK3 kinase inhibitors with the help of AI‐assisted drug design. b. UNITY 2D fingerprint similarity comparison between 332 compounds from the traditional Chinese medicine library and the designed template small‐molecule compound. c. The top 10 compounds are listed based on the ranking of similarity score. d. The in vitro kinase assay showing the changes in the phosphorylation levels of MEK5 following the treatments of the top 10 compounds. Quantification of densitometric ratios for p‐MEK5/MEK5 are shown and the results are representative of three independent experiments. e. Three new small‐molecule compounds were obtained following the structural modifications of DHT. f. Binding affinities of DHT, DPFD, DDNP and DPDH to MEKK3 protein were measured by SPR analysis. g. The binding energies of DHT, DPFD, DDNP and DPDH to MEKK3 protein were predicted using the AutoDockTools. h. The in vitro kinase assay showing changes in the phosphorylation levels of MEK5 following the treatment with DHT, DPFD, DDNP and DPDH. Quantification of densitometric ratios for p‐MEK5/MEK5 are shown and the results are representative of three independent experiments. i. The in vitro kinase assay showing changes in the phosphorylation levels of MEK5 following the treatment with different concentrations of DPDH (2–8 µM). Quantification of densitometric ratios for p‐MEK5/MEK5 are shown and the results are representative of three independent experiments.

Moreover, to further verify the direct interactions between DPDH and MEKK3, the cellular thermal shift assay (CETSA) was performed. It was shown that the thermal stability of MEKK3 was robustly enhanced in the presence of DPDH, suggesting that DPDH was capable of binding to MEKK3 (Figure 3a,b). This was substantiated by the drug affinity responsive target stability (DARTS) assay, which deciphered that there was a reduction in the protease susceptibility of MEKK3 upon the binding of DPDH (Figure 3c,d). Subsequently, molecular docking was conducted to predict DPDH's potential binding site on MEKK3, and it was found that the NH group of DPDH could directly bind to the Asp44 residue of MEKK3 to prohibit its kinase activity (Figure 3e). With the purpose of further defining the function of Asp44 residue of MEKK3 in maintaining the structural stability of DPDH‐MEKK3 binding complex, the 100 ns molecular dynamics simulation was performed to dissected whether DPDH was able to exert an allosteric modulation on MEKK3 protein with D44G mutation. It was elucidated that DPDH was not able to tighten the MEKK3 binding domain and adjust its conformation upon D44G mutation (Figure 3f). The subsequent dynamic stability data emphasized that mutation of Asp44 residue strikingly altered the root mean square deviation (RMSD) values of the DPDH‐MEKK3 binding complex (Figure 3g). More importantly, microscale thermophoresis (MST) analysis unraveled that DPDH was inclined to bind to MEKK3 wild type protein in a dose‐dependent manner (Figure 3h). Conversely, DPDH failed to display the binding affinity to the MEKK3 protein with D44G mutation even with the elevated concentrations (Figure 3i). Further, kinase activity assessment demonstrated that DPDH failed to inhibit the MEKK3 kinase activity upon the D44G mutation in MEKK3, as reflected by the p‐MEK5 levels (Figure 3j). In line with this, DARTS assay illustrated that DPDH lost the binding capability to MEKK3 protein with D44G mutation (Figure 3k,l). As MEKK3 and MEK5 are predisposed to interact via their PB‐1 (Phox and Bem1) domains [24], Co‐IP results demonstrated that DPDH could weaken the MEKK3—MEK5 protein‐protein interaction (Figure S9a). Based on these results, we propose that DPDH binds to the Asp44 residue of MEKK3 and acts as a steric disruptor of MEKK3—MEK5 heterodimerization, thereby diminishing the phosphorylation of MEK5.

**FIGURE 3:**
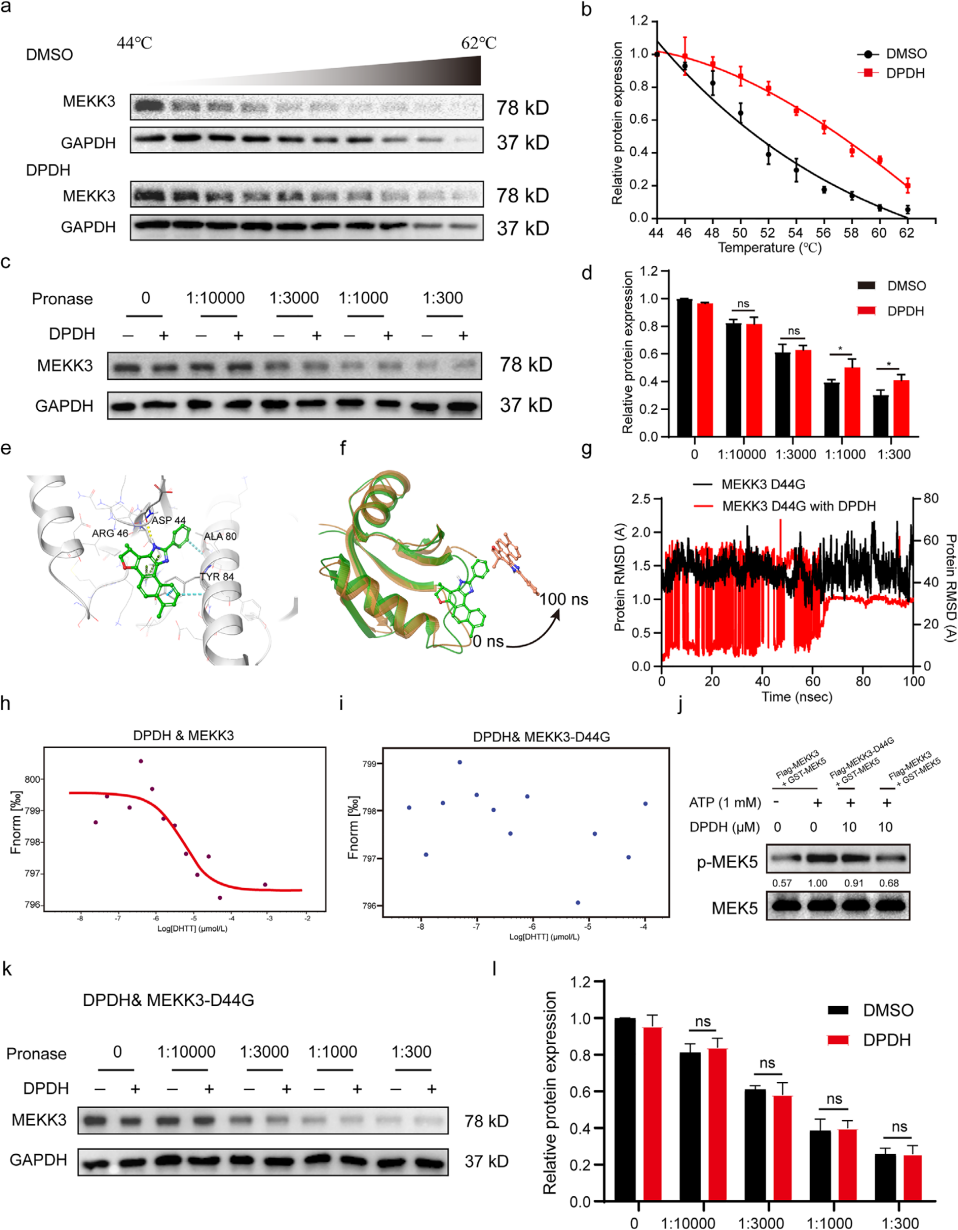
Asp44 residue of MEKK3 is required for its interactions with DPDH. CETSA (a, b) and DARTS (c, d) assays were carried out to examine the direct binding of DPDH to MEKK3 protein, n = 3. e. The potential binding sites of DPDH to MEKK3 protein were predicted by molecular docking. f. Molecular dynamics simulation showing the presentative conformation of MEKK3 with D44G mutation binding with DPDH. g. The time evolution of RMSD values for MEKK3 protein with D44G mutation in the absence or presence of DPDH. MST assay showing the binding affinities of DPDH to MEKK3 wild type protein (h) or MEKK3 protein with D44G mutation (i). j. The in vitro kinase assay showing changes in the phosphorylation levels of MEK5 with MEKK3 wild type protein or MEKK3 protein harboring D44G mutation, in the absence or presence of DPDH. Quantification of densitometric ratios for p‐MEK5/MEK5 are shown and the results are representative of three independent experiments. k, l. DARTS assay was performed to determine the binding affinity of DPDH to MEKK3 protein with D44G mutation, n = 3. The protein expression level of MEKK3 was quantified by densitometric analysis. All data are expressed as mean ± SD. ^*^ *p* <0.05, ns represents not significant.

We next performed a comprehensive kinome‐wide evaluation integrating in silico prediction and experimental validation to test the AI‐guided finding that DPDH effectively inhibits MEKK3. First, we identified 311 potential kinase targets of DPDH through virtual screening against the KIPP database (Figure S9b). We then prioritized 110 kinases closely related to CCM pathogenesis based on compensatory signaling pathways, structural domain homology, and kinase family relationships. Intersection of these datasets yielded 46 candidate kinases, which were subsequently profiled experimentally (Figure S9c). Notably, 10 µM DPDH inhibited MEKK3 by 99.3%—the highest inhibition observed across all kinases tested (Figure S9d). Collectively, these findings provide the rationale for prioritizing MEKK3 in downstream functional validation and establish screening‐level evidence supporting MEKK3 as the primary target of DPDH in the CCM setting.

### 2.3 DPDH Inhibits the Growth of CCM Lesions and Is Prone to Localize at the Lesion Regions

Due to that our above data have implicated that DPDH can act as a potent inhibitor for MEKK3 kinase activity as validated by the inhibited phosphorylation of MEK5, we subsequently investigated whether DPDH could play a role in influencing the progression of CCM lesions. In this sense, 4‐HT was injected into the *Krit1^iECKO^* pups at P1 to induce the formation of CCM lesions, followed by the intraperitoneal injection of DPDH (5 mg/kg or 10 mg/kg) or DHT (10 mg/kg) at P11, P13, P15 and P17. The CCM lesion burden was analyzed at P19 (Figure 4a). Noteworthily, DPDH (10 mg/kg) markedly alleviated the burden of CCM lesions in the hindbrains, as validated by the visual inspection photos. Likewise, H&E staining analysis unveiled that *Krit1^iECKO^* mice had high propensity to develop CCM lesions with dilated and enlarged capillaries in the cerebella compared to the *Krit1^fl/fl^* mice, whereas the treatment with 10 mg/kg of DPDH contributed to prominent reduction in the size of CCM lesions in the *Krit1^iECKO^* mice (Figure 4b). To precisely quantify the formation of CCM lesions, high‐resolution microCT was utilized to image the P19 hindbrains and the actual lesion volumes were examined by semi‐automated software. Of note, the administration of 10 mg/kg DPDH extensively attenuated the number of developed lesions seen in the hindbrains of *Krit1^iECKO^* mice, which showed the superior therapeutic effect than the treatment of DHT (10 mg/kg) (Figure 4c,e). We next classified the formed CCM lesions on the basis of their size, and found conspicuously diminished number of large‐sized (>10^7^ µm^3^), medium‐sized (10^6^ to 10^7^ µm^3^) as well as small‐sized CCM lesions (<10^6^ µm^3^) in the *Krit1^iECKO^* mice following the treatment of 10 mg/kg of DPDH (Figure 4f). Meanwhile, treatment of *Krit1^iECKO^* mice with 10 mg/kg DPDH resulted in a profound decrease in terms of the volumes of CCM lesions in comparison to the vehicle control (Figure 4g). Further, in the retinas of *Krit1^iECKO^* mice, IB4 staining highlighted that venous malformations were inclined to develop at the periphery of vascular plexus, which could be mitigated in response to the treatment of 10 mg/kg DPDH (Figure 4d,h and Movies 3–7). To further characterize the pharmacological effect of DPDH in the *Ccm2^iECKO^* mice, the same treatment regimen was applied (Figure S10a). Similar to the results observed in the *Krit1^iECKO^* mice, 10 mg/kg of DPDH was capable of strikingly ameliorating the CCM lesion burden in the *Ccm2^iECKO^* mice (Figure S10b). Together, these data illuminate that DPDH can profoundly retard the progression of CCM lesions.

**FIGURE 4:**
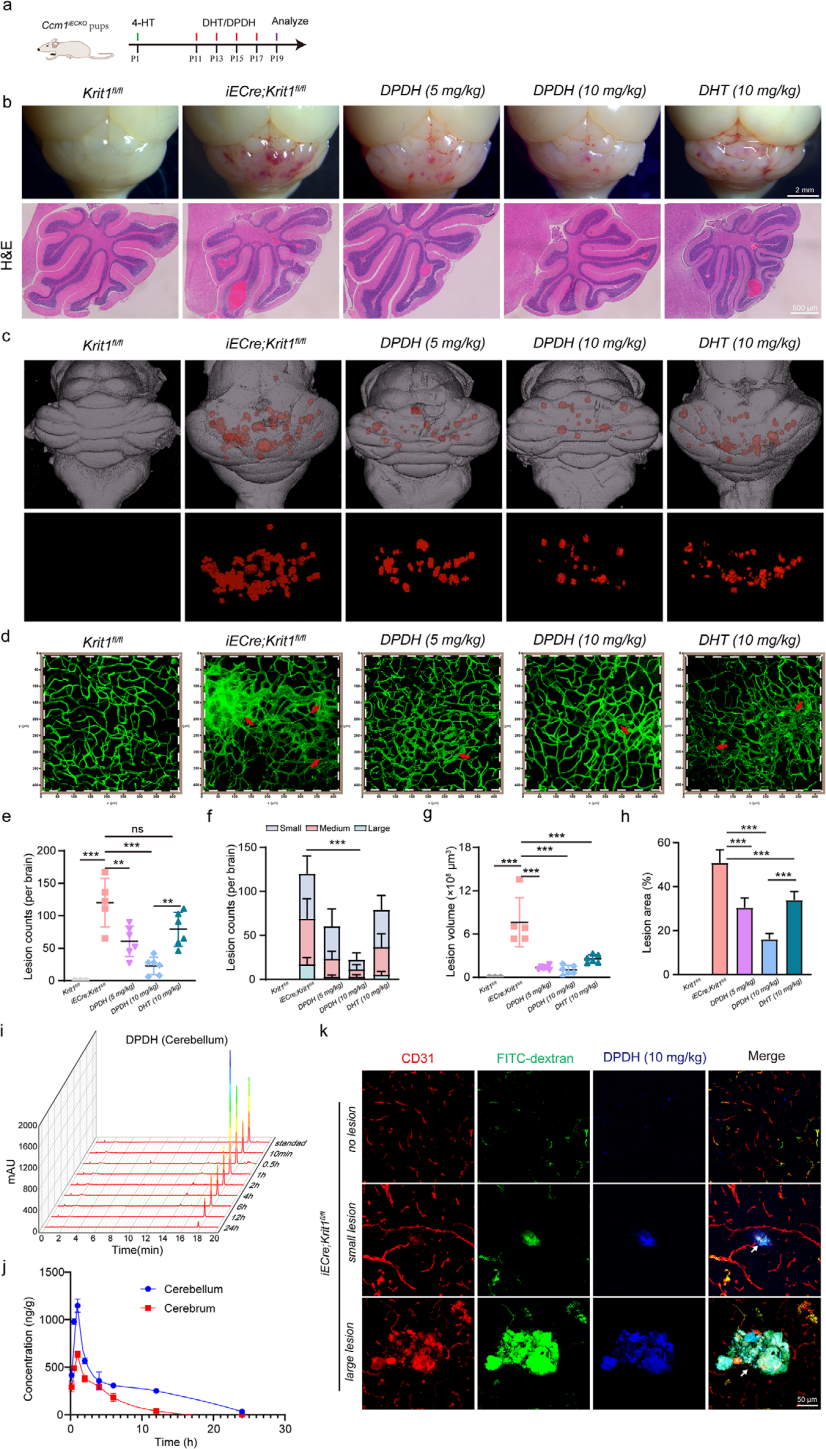
DPDH inhibits the growth of CCM lesions and is prone to localize at the lesion regions. a. Schematic diagram of experimental design. b. Top panel: visual appearance of CCM lesions in the hindbrains of mice in different groups, n = 6–10. Scale bar: 2 mm. Bottom panel: representative images of H&E staining in different groups are shown. Scale bar: 500 µm. c. Representative microCT images of CCM lesions in different groups. CCM lesions are shown in red, n = 3–6. d. IB4 staining of retinal blood vessels in different groups, n = 5. Arrows indicate lesions. e‐g. Quantification of microCT analysis from different perspectives. h. Quantification of lesion areas in the retinal blood vessels (refer to d). i. Representative LC‐MS chromatograms of DPDH at different time points in the cerebella of *Krit1^iECKO^* mice. j. Concentration‐time curves of DPDH in the cerebra and cerebella of *Krit1^iECKO^* mice following a single injection of DPDH (10 mg/kg, i.p.), n = 3. k. Representative immunofluorescence images of DPDH distribution in the cerebella of *Krit1^iECKO^* mice at the 0.5 h time point following a single injection of DPDH (10 mg/kg, i.p.), n = 3. CD31 staining (red) denotes blood vessels, FITC‐dextran is labelled in green, and blue color represents the intrinsic fluorescence of DPDH. Scale bar: 50 µm. All data are expressed as mean ± SD. ^**^ *p* <0.01, ^***^ *p* <0.001, ns represents not significant.

In addition, to exploit the pharmacokinetic profiles of DPDH in the brains of *Krit1^iECKO^* mice, we examined the concentrations of DPDH in both cerebrum and cerebellum by liquid chromatography‐mass spectrometry (LC‐MS). The cerebrum and cerebellum tissue samples were collected at 10 min, 0.5 h, 1 h, 2 h, 4 h, 6 h, 12 h, and 24 h after administration of DPDH. Of interest, the concentration of DPDH was able to reach the peak 1 h following the intraperitoneal injection of DPDH (10 mg/kg) (Figure 4i,j). Furthermore, the obtained concentration‐time curves showed that the concentration of DPDH in the cerebellum where the lesions tend to occur was approximately 2.4‐fold higher than that in the cerebrum (no lesion), implying that DPDH was prone to locate at the lesion regions (Figures 4j and S10c,d). The in vivo pharmacokinetic parameters uncovered that DPDH by intraperitoneal administration possessed acceptable metabolic stability and bioavailability (Figure S10d). Meanwhile, taking advantage of the intrinsic fluorescent property of DPDH, we revealed that the detected DPDH in the lesion regions was enriched with the increasing size of lesions, as compared to the areas without lesions in the cerebellum (Figure 4k). In comparison to DPDH, DHT was barely detectable at the 6 h time point, suggesting that DHT exhibited relatively short duration in the brains and its distribution in the cerebellum and cerebrum appeared not to be biased from 2 h after the injection (Figure S10e–g). Further, no acute toxicity was observed in mice treated with DPDH (upto 320 mg/kg), as evidenced by the absence of mortality and no discernable histopathological abnormalities in major organs, validating the safety of DPDH (Figure S10h). Overall, our findings manifest that DPDH serves as a BBB‐penetrant therapeutic candidate with favorable pharmacokinetics and safety profile, exerting a compelling effect on limiting the progression of CCM lesions.

### 2.4 DPDH Corrects the Aberrant Blood Vessels and Improves Vascular Integrity in CCM Lesions

It has been well established that CCM lesions are featured with damaged endothelial junctions as well as disrupted EC‐pericyte interactions [25]. Firstly, we obtained four clinical specimens of CCM patients from the Sun Yat‐Sen University Cancer Center, it was found that the samples contained typical CCM lesions as well as surrounding relatively normal tissues on the basis of the H&E staining results (Figure 5a). Further, in the lesions of collected CCM patients, it was observed that the distribution of AJs in the blood vessels of lesions was conspicuously disrupted compared with that in the normal vessels, as visualized by the VE‐cadherin (VE‐cad) staining. Meanwhile, the blood vessels in the lesional regions also displayed broken TJs, which was substantiated by the ZO‐1 staining, when compared to the surrounding normal blood vessels (Figures 5b and S11a). Since we have illustrated that DPDH plays a critical role in limiting the growth of already established CCM lesions, we next evaluated whether DPDH could exert any impacts on regulating the vascular structure in the lesions. In this basis, we examined the blood vessels in lesional regions in detail after the treatment of DPDH, and it was elucidated that, in the *Krit1^iECKO^* mice, DPDH was capable of boosting the expression levels of endothelial junctions‐associated proteins including Claudin‐5 and VE‐cad in the blood vessels of lesional regions, thereby improving the junctional integrity (Figure 5c–e). Additionally, co‐immunostaining of CD31 and NG2 highlighted that the coverage of pericytes along the blood vessels in the lesional area was robustly augmented following the administration of DPDH (Figure 5c,f). Of note, in comparison to the *Krit1^fl/fl^* mice, basal membrane (Collagen IV) around the blood vessels in CCM lesions presented compensatory thickening in the *Krit1^iECKO^* mice, which was congruent with previous report [26]. When subjected to DPDH treatment, the basement membrane support around the blood vessels in the lesional regions was restored accordingly (Figure 5c,g). Similar phenomena were observed in the retinal blood vessels, which showed that DPDH was able to reverse the aberrant retinal vasculature of CCM lesions (Figure S11b). Further, images taken by SEM uncovered that the *Krit1^iECKO^* and *Ccm2^iECKO^* mice received 10 mg/kg of DPDH exhibited normalized vascular endothelium with strengthened EC junctions and fortified morphology of inner layer (Figures 5h and S11c). Disruption of vascular integrity promotes tissue factor‐driven thrombin activation, converting soluble fibrinogen into insoluble fibrin monomers that assemble into cross‐linked clots with activated platelets and erythrocytes. Consistent with this pathogenic cascade, CD41 (a platelet marker) co‐localized with fibrinogen alpha chain in the lesional regions of *Krit1* ^iECKO^ mice, indicative of intravascular thrombosis (Figure S11d). Notably, DPDH robustly decreased the expression levels of CD41 and fibrinogen alpha chain in the *Krit1* ^iECKO^ mice, suggesting that DPDH attenuates thrombus formation (Figure S11d–f).

**FIGURE 5:**
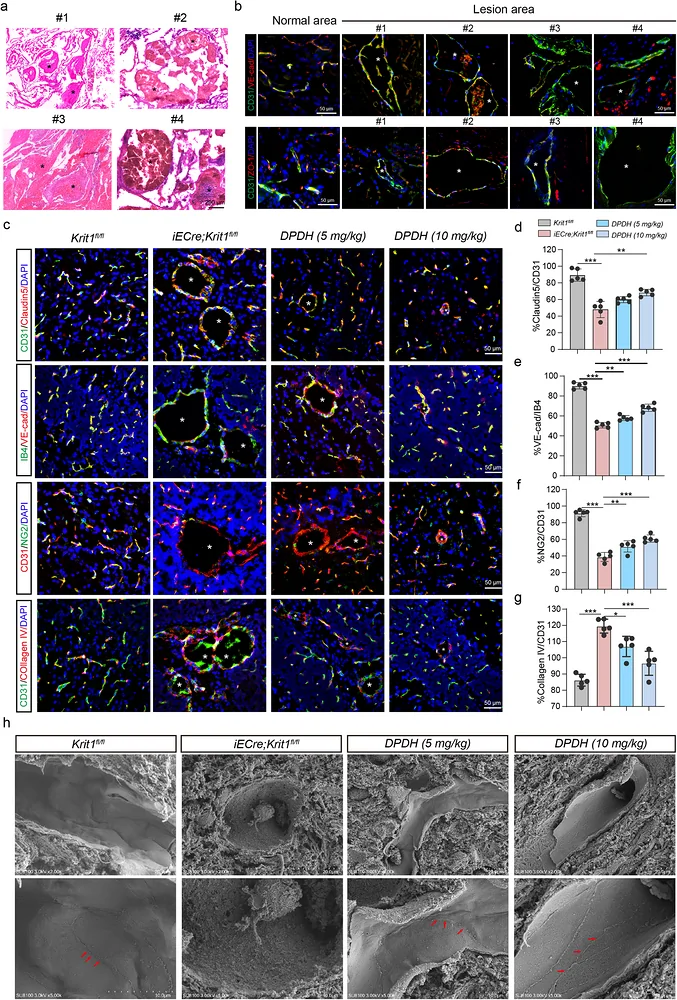
DPDH normalizes the vascular structure of CCM lesions in the *Krit1^iECKO^* mice. a. Representative H&E staining images of the four clinical specimens of CCM patients. Scale bar: 200 µm. b. Representative co‐immunofluorescence staining images for CD31/VE‐cad/DAPI (top panel) and CD31/ZO‐1/DAPI (bottom panel) in the blood vessels of both normal area and lesional area in the human CCM specimens, n = 4. Scale bar: 50 µm. c. Representative co‐immunofluorescence staining images for CD31/Claudin‐5/DAPI, CD31/VE‐cad/DAPI, CD31/NG2/DAPI, and CD31/Collagen IV/DAPI in the blood vessels of the cerebella harvested from the *Krit1^iECKO^* mice in the absence or presence of DPDH treatment, n = 5. Asterisks indicate lesions. Scale bar: 50 µm. Quantification for the percentages of Claudin‐5 (d), VE‐cad (e), NG2 (f) and Collagen IV (g) coverage on CD31^+^ blood vessels, n = 5. h. Representative SEM images for the blood vessels in the hindbrains harvested from the mice in different groups, n = 3. EC junctions are indicated by red arrows. Scale bars: 20 µm (top panel) and 10 µm (bottom panel). All data are expressed as mean ± SD. ^*^ *p* <0.05, ^**^ *p* <0.01, ^***^ *p* <0.001.

It has been widely appreciated that pathological dilations visualized in blood vessels of CCM lesions are prone to give rise to turbulent, low‐blood flow that aggravates endothelial dysfunction, inflammation and even ruptures [27]. At present, the advanced approaches of monitoring blood flow on a brain‐wide scale have achieved great progress. Herein, functional ultrasound was employed to perform large‐scale brain imaging. Intriguingly, the results demonstrated that the density and speed of blood flow in the lesional regions of *Krit1^iECKO^* mice appeared to be much slower than that of *Krit1^fl/fl^* mice, but the decreased density and speed of blood flow could be rescued in the presence of 10 mg/kg DPDH (Figure 6a). Consistently, the analysis of regional cerebral blood flow (CBF) by laser speckle contrast imaging system exhibited similar phenotypes (Figure S11g). Since the unique feature of brain blood vessels is the BBB produced by the brain neurovascular unit, we then interrogated whether DPDH had any consequences on influencing the function of BBB in the *Krit1^iECKO^* mice. It was shown that DPDH blunted the vascular permeability of the lesions in both the cerebellar (Figure 6b) and retinal blood vessels, as evidenced by the leakage of EBD (Figure 6c,f). More interestingly, FITC‐dextran was found to be restricted inside capillary beds of brains in the *Krit1^fl/fl^* mice, but was diffused to surrounding tissues in the cerebella of *Krit1^iECKO^* mice. The treatment of DPDH considerably impeded the leakage of FITC‐dextran from local brain vasculature, implying the attenuated vascular permeability under the exposure of DPDH (Figure 6d,g,h). Similar phenotypes were observed in the *Ccm2^iECKO^* mice (Figure S11h,i). In order to visualize the CCM microvessels in a 3D manner, the cerebella were dissected from the *Krit1^iECKO^* mice after the injection of FITC‐dextran, and the blood vessels were then analyzed using the two‐photon microscopy. We delineated that DPDH (10 mg/kg) intervention was capable of mitigating the dextran leakage in the *Krit1^iECKO^* mice and decreasing the diameter of inflated veins (Figure 6e,i,j and Movies 8–10). Based on these data, we conclude that DPDH corrects aberrant vascular structure, improve blood flow and normalize EC barrier function in the CCM mouse models.

**FIGURE 6:**
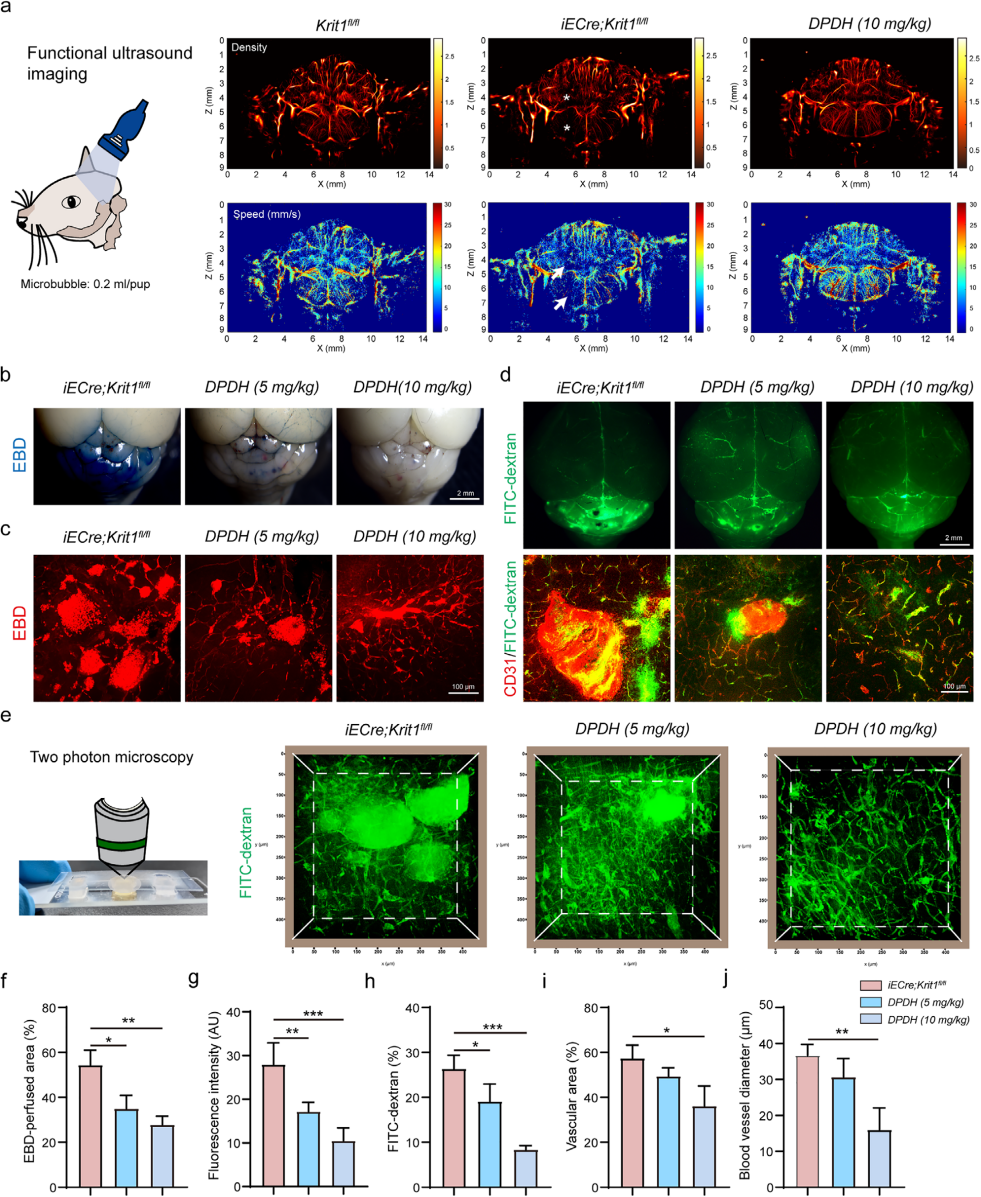
DPDH improves the blood flow and normalizes the EC barrier function in the CCM lesions of *Krit1^iECKO^* mice. a. Representative functional ultrasound images of the density (top panel) and speed (bottom panel) of blood flow in the cerebella harvested from *Krit1^ECKO^* mice in the absence or presence of DPDH treatment. Asterisks indicate lesions. b. Representative visual inspection photos of brains upon the administration of EBD in the *Krit1^iECKO^* mice in the absence or presence of DPDH treatment, n = 4–6. Scale bar: 2 mm. c. Representative immunofluorescence images of retinal vasculature upon the administration of EBD in the *Krit1^iECKO^* mice in the absence or presence of DPDH treatment, n = 3. Scale bar: 100 µm. d. Representative fluorescence images of brain tissues (top panel) and representative immunofluorescence images of frozen sections stained with CD31 (bottom panel) following the administration of FITC‐dextran in the *Krit1^iECKO^* mice in the absence or presence of DPDH treatment, n = 3–5. Scale bars: 2 mm (top panel) and 100 µm (bottom panel). e. Representative two‐photon microscopy images of vascular malformations after injection of FITC‐dextran in the absence or presence of DPDH treatment, n = 3. f. Quantification of EBD‐perfused area (refer to c). n = 3. g. Quantification for the fluorescence intensity of FITC‐dextran (refer to d top panel), n = 3–5. h. Quantification of FITC‐dextran‐perfused area (refer to d bottom panel), n = 3. i. Quantification of vascular area (refer to e), n = 3. j. Quantification of blood vessel diameter (refer to e), n = 3. All data are expressed as mean ± SD. ^*^ *p*<0.05, ^**^ *p* <0.01, ^***^ *p* <0.001.

### 2.5 DPDH Restricts the Activation of MEKK3‐KLF2/4‐pMLC2 Signaling Conferred by CCM Gene Knockdown in ECs

It has been established that deletions of CCM genes in ECs give rise to the formation of CCM lesions through activating MEKK3‐KLF2/4 signaling cascade. Therefore, we next explored whether DPDH could rescue the phenotypes of CCM lesions by virtue of modulating the aberrant MEKK3 downstream signaling. Strikingly, the immunofluorescence staining data showed that DPDH co‐localized with MEKK3 in both *Krit1^iECKO^* mice and si‐*KRIT1*‐hCMEC/D3 cells, further validating the inhibitory effect of DPDH on MEKK3 (Figures 7a and S12a,b). The following western blot analysis demonstrated that the elevated expression levels of KLF2 and KLF4 upon knockdown of *KRIT1* gene in the hCMEC/D3 cells (*sh‐KRIT1‐*hCMEC/D3) could be reversed following the treatment of 4 µM or 8 µM DPDH, which did not have significant impacts on the viability of *sh‐KRIT1‐*hCMEC/D3 cells (Figures 7b,c and S12c,d). In concomitant with the above in vitro data, DPDH was able to exert a striking effect on attenuating the expression level of KLF2 in the vascular endothelium of CCM lesions in the *Krit1^iECKO^* mice (Figure 7d,f). As the activated MEKK3 signaling pathway yields augmented phosphorylation of MEK5, in turn, contributes to hyperphosphorylation of ERK5, we further determined the expression level of phosphorylated ERK5 following the treatment of DPDH. It was found that the phosphorylation of ERK5 was dramatically boosted upon the knockdown of *KRIT1* gene, but this increase in ERK5 phosphorylation was remarkably impaired in response to DPDH treatment (Figure 7b,c). Despite that P38, JNK and ERK1/2 are thought to be the downstream targets of MEKK3 [28, 29, 30], DPDH exerted no compelling effects on the phosphorylation levels of P38, JNK and ERK1/2 (Figure S12f).

**FIGURE 7:**
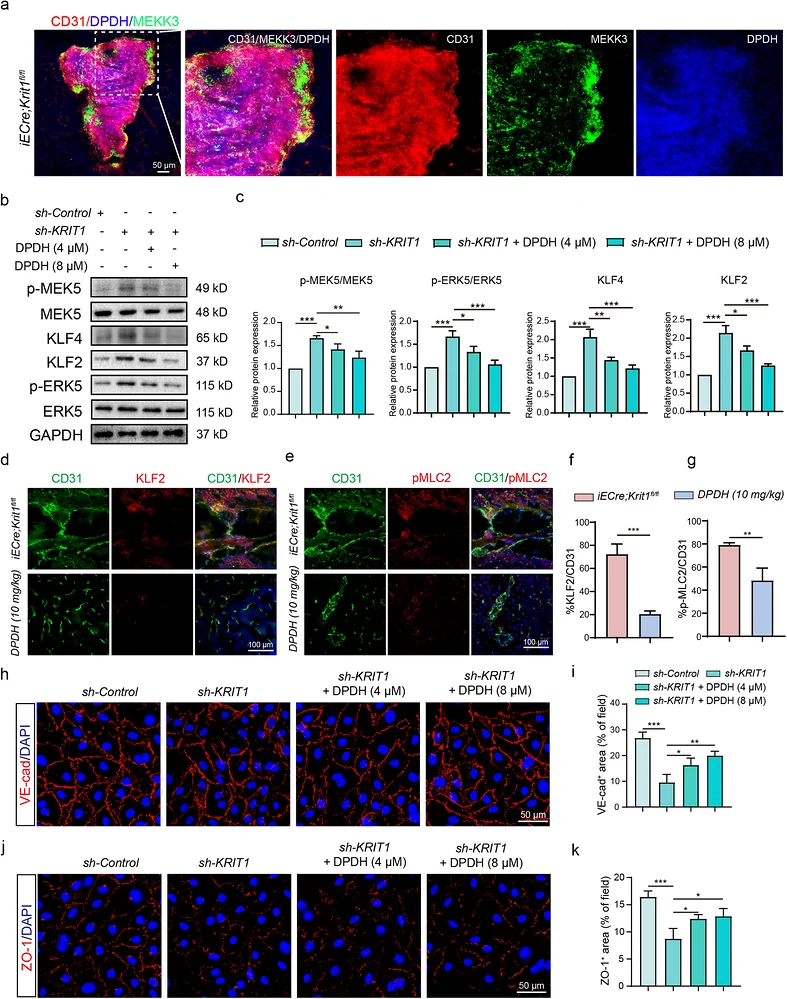
DPDH restricts the activation of MEKK3‐KLF2/4‐pMLC2 signaling conferred by CCM gene knockdown in ECs. a. Representative immunofluorescence staining images for CD31/MEKK3/DPDH in the cerebella of *Krit1^iECKO^* mice 0.5 h following a single injection of DPDH (10 mg/kg, i.p.), n = 3. Scale bar: 50 µm. The region highlighted in a white dotted box is magnified. b. Western blot analysis showing the changes in the protein expression levels of KLF2, KLF4, MEK5, p‐MEK5, ERK5 and p‐ERK5 with or without the treatment of DPDH in the sh‐*KRIT1*‐hCMEC/D3 cells, GAPDH was used as a loading control. c. Quantification of densitometric ratios for the protein expression levels of KLF2, KLF4, p‐MEK5/MEK5, and p‐ERK5/ERK5, n = 3. d. Representative immunofluorescence staining images for CD31 and KLF2 in the *Krit1^iECKO^* mice in the absence or presence of DPDH treatment. Scale bar: 100 µm. e. Representative immunofluorescence staining images for CD31 and pMLC2 in the *Krit1^iECKO^* mice in the absence or presence of DPDH treatment. Scale bar: 100 µm. f. Quantification for the percentage of KLF2 (d) coverage on CD31^+^ blood vessels, n = 3. g. Quantification for the percentage of pMLC2 (e) coverage on CD31^+^ blood vessels, n = 3. h. Representative immunofluorescence images for VE‐cad (red)/DAPI (blue) in the HUVECs. Scale bar: 50 µm. i. Quantification for the percentage of VE‐cad^+^ area in the visual field, n = 3. j. Representative immunofluorescence images for ZO‐1 (red)/DAPI (blue) in the HUVECs. Scale bar: 50 µm. k. Quantification for the percentage of ZO‐1^+^ area in the visual field, n = 3. All data are expressed as mean ± SD. ^*^ *p*<0.05, ^**^ *p* <0.01, ^***^ *p* <0.001.

Furthermore, it has been argued that Rho signaling, as an alternative effector pathway located at the downstream of MEKK3‐KLF2/4 signaling cascade, may play a pivotal role in driving the pathogenesis of CCM lesions. To further inspect whether DPDH was capable of exerting any impacts on the abnormal activation of Rho signaling triggered by the deletions of CCM genes, we thus performed the immunofluorescence staining of pMLC2 that serves as a downstream effector of Rho signaling. It was revealed that the administration of DPDH was able to diminish the level of pMLC2 in the CCM lesions of *Krit1^iECKO^* mice (Figure 7e,g). Consistent results were further observed in the *sh‐KRIT1*‐hCMEC/D3 cells (Figure S12g). Likewise, boosted Rho activity has been reported to be closely associated with reinforced formation of stress fibers and further loosened junctions in ECs as well as damaged vascular integrity in mice [31]. Accordingly, we sought to investigate the effects of DPDH on endothelial permeability and junction‐related proteins. DPDH significantly increased transendothelial electrical resistance (TEER) values, decreased FITC‐dextran leakage and elevated the expression levels of VE‐cad and ZO‐1 in the sh‐*KRIT1*‐HUVECs (Figures 7h,k and S12h–j). Hence, these findings indicate that DPDH strengthens vascular integrity via inhibiting the transduction of MEKK3‐KLF2/4‐pMLC2 signaling.

### 2.6 DPDH Attenuates Microglial Uptake of Endothelial Debris by Inhibiting the MEKK3/NF‐κB Axis

To better demonstrate the underlying mechanisms of DPDH retarding the progression of CCM lesions, we thus carried out the RNA‐seq to examine the transcriptome of ECs isolated from the cerebella of P19 *Krit1^iECKO^* mice in the absence or presence of DPDH treatment (Figure 8a). Bioinformatics analysis of differentially expressed genes (DEG) identified multiple enriched GO terms related to angiogenesis and endothelial inflammation, including regulation of acute inflammatory response, blood vessel diameter maintenance, endothelial cell proliferation, and ameboidal‐type cell migration (Figure 8b). It has been gradually recognized that inflammatory response plays a critical role in aggravating the formation of brain vascular malformations in both human and mouse CCM models [32, 33]. This raised a question of whether modulation of immune cells could be involved in the ameliorated CCM phenotypes challenged by DPDH. Of interest, it was shown that a series of representative genes including *Ccl11*, *Ccl19* and *Adamts4* were down‐regulated according to the RNA‐seq results (Figure 8c). In coincidence with the RNA‐seq findings, qRT‐PCR data verified that DPDH precipitated pronounced reduction in the mRNA expression levels of *Ccl11*, *Ccl19* and *Adamts4* in the ECs isolated from *Krit1^iECKO^* mice (Figures 8d and S13a). Since proteolytic activity of ADAMTS as effector downstream of MEKK3‐KLF2/4 signaling has been reported to aggravate the formation of CCM lesions [34], this observation partially implicated that DPDH halted the development of CCM lesions via antagonizing MEKK3 kinase activity. Meanwhile, both CCL11 and CCL19 have been documented to participate in biological behaviors of immune cells [35, 36], we therefore examined the potential role of DPDH in modulating immune cells in CCM lesions. DPDH diminished the amounts of CD45^+^ leukocytes, CD4^+^ T cells and microglial cells (CD11b^+^CD45^low/int^), but failed to influence the number of CD8^+^ T cells isolated from the *Krit1^iECKO^* mice (Figures S13b–e and S15).

**FIGURE 8:**
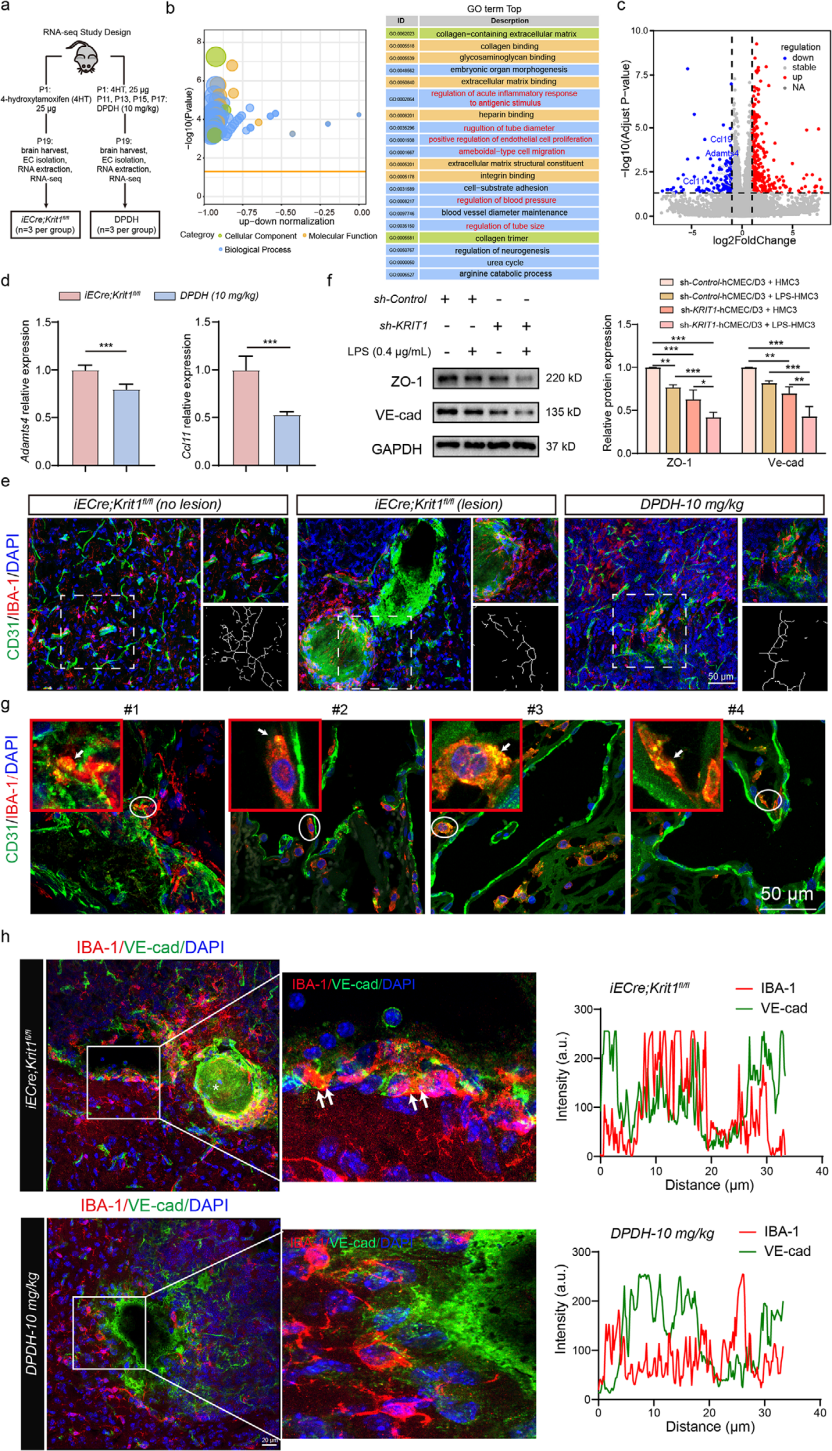
DPDH retards the microglia uptake of damaged EC debris in the *Krit1^iECKO^* mice. a. Schematic diagram of the experimental design for transcriptomic study in the *Krit1^iECKO^* mice in the absence or presence of DPDH treatment. b. RNA‐Seq analysis of DEG showing the top 20 most enriched GO terms in the *Krit1^iECKO^* mice following the treatment of DPDH. c. Volcano plot showing the DEG following the treatment of DPDH treatment. d. Quantification of the mRNA expression levels for *Adamts4* and *Ccl11* in the ECs isolated from *Krit1^iECKO^* mice in the absence or presence of DPDH treatment, n = 5. e. Representative co‐immunofluorescence staining for CD31 and IBA‐1 in the cerebella dissected from the *Krit1^ECKO^* mice, n = 3. Scale bar: 50 µm. Top right: the region highlighted in white dotted boxes are magnified and the white arrows indicate co‐localization of IBA‐1 and CD31. Bottom right: microglial cells were visualized and the processes were traced by ImageJ software. f. Western blot analysis of ZO‐1 and VE‐cad protein expression levels in the hCMEC/D3 cells co‐cultured with HMC3 cells, GAPDH was used a loading control, n = 3. g. Representative co‐immunofluorescence staining for CD31 (ECs) and IBA‐1 (microglial cells) in the human CCM specimens, n = 4. Scale bar: 50 µm. The regions highlighted in white circles are magnified and the white arrows indicate that microglial cells phagocytized the endothelium. h. Left: representative immunofluorescence staining for VE‐cad and IBA‐1 in the cerebella dissected from the *Krit1^iECKO^* mice, n = 3. Scale bar: 20 µm. The regions highlighted in white boxes are magnified and the white arrows indicate that microglial cells phagocytized the endothelium. Right: representative co‐localization analysis of IBA‐1 and VE‐cad in the *Krit1^iECKO^* mice. All data are expressed as mean ± SD. ^*^ *p*<0.05, ^**^ *p* <0.01, ^***^ *p* <0.001.

Notably, immunofluorescence staining revealed that the number of microglial cells was markedly increased in the mature lesions compared with that in the initial lesions (Figure S13f–g). Microglial depletion using PLX5622 significantly suppressed CCM progression and impeded FITC‐dextran leakage, indicating that microglial cells actively contribute to CCM pathogenesis (Figure S13h–m). Microglial cells in lesional regions were activated, displaying markedly shorter processes and enlarged bodies in comparison to the non‐lesional areas in the *Krit1^iECKO^* mice (Figure 8e). To simulate the inflammatory condition in the *Krit1^iECKO^* mice, microglial cells were activated with the stimulation of lipopolysaccharide (LPS), as supported by the elevated mRNA expression levels of *IL‐6* and *IL‐1β* in the HMC3 cells (Figure S13n). Next, we co‐cultured sh‐*KRIT1*‐hCMEC/D3 cells with LPS‐treated HMC3 cells to explore the interactions between CCM endothelium and activated microglial cells. Western blot analysis revealed that, in comparison to the *sh*‐*Control*‐hCMEC/D3 + LPS‐HMC3 group, KRIT1 silenced ECs upon co‐culture with LPS‐stimulated microglial cells exhibited declined expression of VE‐cad and ZO‐1, implying that activated microglial cells tended to exacerbate the disrupted CCM endothelial integrity (Figure 8f). Additionally, immunofluorescence staining analysis on clinical CCM samples uncovered those microglial cells located at the lesional sites tended to contain the staining of endothelial marker CD31 in the cytoplasm (Figures 8g and S13o), which manifested that microglial cell at the lesional areas could exacerbate endothelial damage via uptake of damaged EC debris. Consistently, co‐immunostaining for IBA‐1 and VE‐cad revealed that IBA‐1^+^ microglial cells intimately associated with and enveloped VE‐cad^+^ endothelial structures at the lesion periphery, indicative of microglial uptake on damaged EC material (Figures 8h and S13p). It was observed that IBA‐1^+^ microglial cells contained CD31^+^ EC debris within the CD68^+^ phagolysosomes in the *Krit1^iECKO^* mice (Figure S13q). All of these phenotypes could be rescued in response to DPDH treatment. Meanwhile, we performed the flow cytometry analysis and detected a distinct population of CX3CR1^+^CD31^+^ cells in *Krit1^iECKO^* mice, suggesting microglial uptake of EC debris. More essentially, treatment with DPDH significantly decreased the percentage of CX3CR1^+^CD31^+^ cell population (Figure S13r,s). Overall, these findings indicate that lesion‐associated microglia tend to remain activated and exhibit increased uptake of damaged EC debris, which is effectively attenuated by DPDH.

It has been appreciated that LPS leads to the activation of intracellular signals via the Toll‐like receptor 4 (TLR4), and depletion of MEKK3 in fibroblasts fail to activate the downstream signaling responses to LPS [37]. Likewise, LPS triggers the activation of MEKK3, allowing macrophages to release IL‐6 and GM‐CSF [22]. To address whether the activation of MEKK3 in the microglial cells results in EC debris uptake, hCMEC/D3‐GFP cells were co‐cultured with Dil‐labeled HMC3 cells and then flow cytometry was employed to determine microglial uptake of damaged EC debris. Notably, LPS‐stimulated HMC3 cells exhibited markedly increased uptake of EC debris compared with non‐stimulated controls. Moreover, silencing of MEKK3 in HMC3 cells dramatically reduced their ability to take up damaged EC debris (Figures 9a and S14a). Meanwhile, DPDH‐treated microglial cells showed diminished capacity to damage the endothelium, suggesting that DPDH suppresses MEKK3 kinase activity to restrict EC debris uptake (Figure 9a,b). To directly visualize microglial uptake of damaged EC debris in vitro, the EC debris were then labeled with carboxyfluorescein succinimidyl ester (CFSE) dye at 37°C for 10 min, followed by that the labeled debris were incubated with microglial cells for 24 h (Figure 9c). Representative high‐resolution images clearly showed that MEKK3 knockdown in HMC3 cells or DPDH treatment significantly inhibited the endothelial fragments within microglial cells (Figure 9d,e). In addition, flow cytometry analysis uncovered that microglial uptake of damaged EC debris was independent of M1/M2 polarization (Figure S14b). ELISA quantification revealed markedly elevated secretion of IL‐1β and IL‐6 in the conditioned medium of the HMC3+LPS+EC debris group relative to the HMC3+LPS group, indicating that damaged EC debris further amplifies the inflammatory response in activated microglia. This effect was significantly suppressed by either MEKK3 knockdown in HMC3 cells or DPDH treatment (Figure S14c). Notably, Microglia‐specific knockdown of MEKK3 was achieved by AAVphp.eb‐Iba1‐Cre‐mediated delivery of shMEKK3 (Figure S14d). Microglial MEKK3 deficiency significantly attenuated CCM progression and mitigated vascular permeability, as evidenced by reduced lesion burden and FITC‐dextran leakage (Figure 9f–i).

**FIGURE 9:**
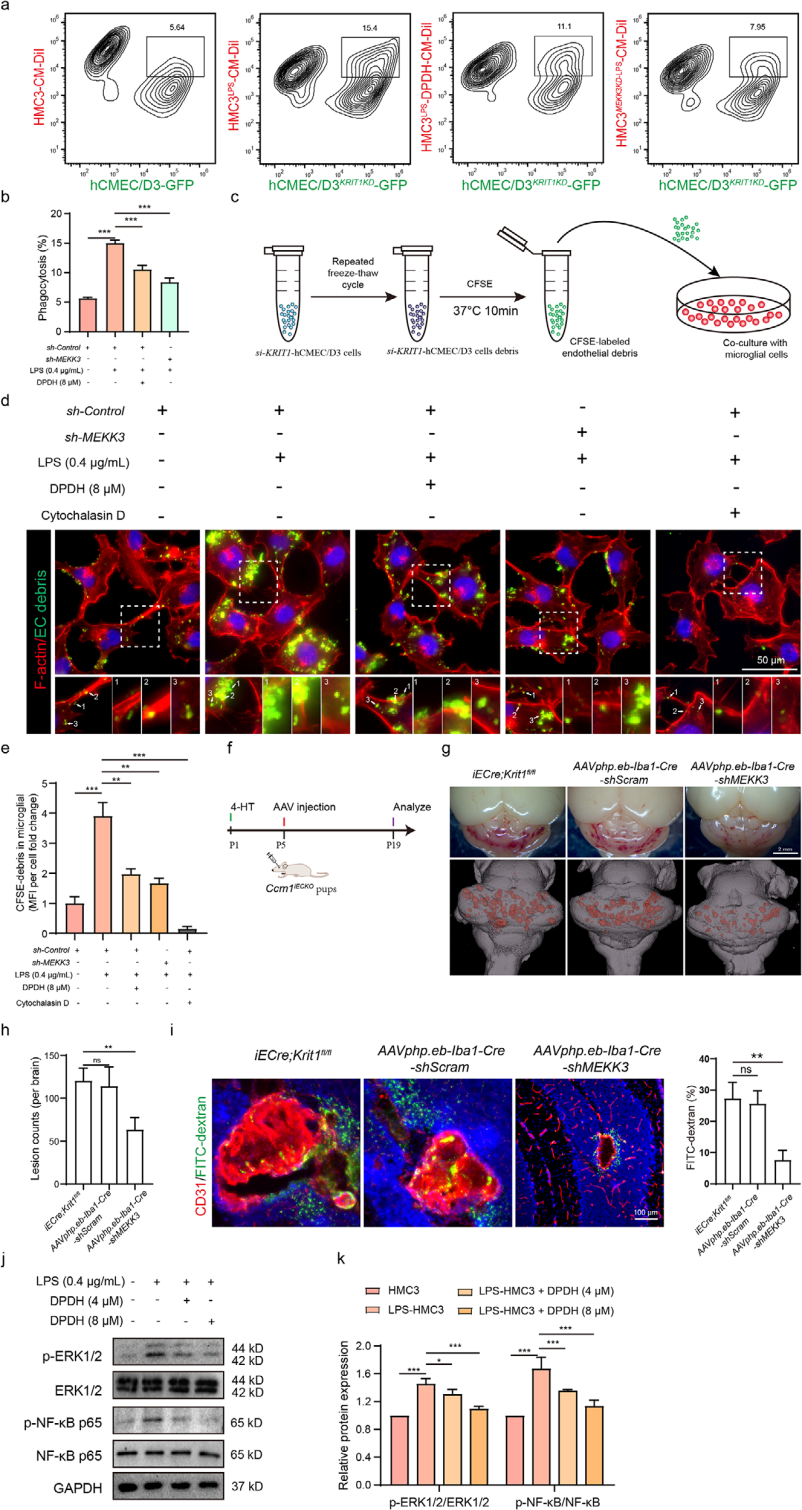
DPDH attenuates microglial uptake of damaged EC debris by inhibiting the MEKK3/NF‐κB axis. a. the hCMEC/D3^GFP^ cells were co‐cultured with Dil‐labeled HMC3 cells in vitro for 24 h, and the total cells were collected. Then, flow cytometry analysis for the phagocytosis of Dil‐labelled hCMEC/D3 cells by HMC3 cells. b. Quantification for the phagocytosis of hCMEC/D3 cells by HMC3 cells, n = 3. c. Schematic diagram of the microglial engulfment assay using CFSE‐labeled EC debris. d. Representative immunofluorescence images displaying microglial engulfment of EC debris in different groups. The regions highlighted in white dotted boxes are magnified on the left; the right images show EC debris approaching microglial cells without contact (1), initiating touch (2), or being internalized (3). Scale bar: 50 µm. e. Quantification for phagocytosis in different groups, n = 3. f. Schematic diagram of experimental design. g. Representative visual inspection and microCT images of CCM lesions in different groups. CCM lesions are shown in red, n = 3. h. Quantification of microCT analysis in different groups. i. Left: representative immunofluorescence images of frozen sections stained with CD31 following the administration of FITC‐dextran in the *Krit1^iECKO^* mice in different groups, n = 3. Scale bar: 100 µm. Right: quantification of FITC‐dextran‐perfused area. j. Western blot analysis for the protein expression of p‐ERK1/2, ERK1/2, p‐NF‐κB p65 and NF‐κB p65 in the HMC3 cells treated with LPS and/or DPDH, GAPDH was used as a loading control, n = 3. k. Quantification of densitometric ratios for the protein expression levels of p‐ERK1/2 and p‐NF‐κB p65, n = 3. All data are expressed as mean ± SD. ^*^ *p*<0.05, ^**^ *p* <0.01, ^***^ *p* <0.001, ns represents not significant.

We next investigated the mechanisms by which DPDH targets MEKK3 to regulate microglial activation. The immunofluorescence staining results elucidated that DPDH could also co‐localize with MEKK3 in the LPS‐stimulated HMC3 cells (Figure S14e). Intriguingly, DPDH robustly diminished the phosphorylation levels of NF‐κB and ERK1/2 (Figure 9j,k), whereas the phosphorylation levels of ERK5, JNK, and P38 remained unaffected in response to DPDH treatment (Figure S14f–i). Co‐immunofluorescence staining also revealed significant co‐localization of IBA‐1^+^ microglial cells with various pro‐inflammatory mediators IL‐6 and IL‐1β (key downstream effectors of MEKK3‐NF‐κB signaling) in the human CCM resections (Figure S14j,k).

Subsequently, we took advantage of a CCM mouse model with more aggravated lesion burden via genetic knockout of CCM2L (CCM2‐like) in the Ccm2‐depleted neonatal mouse brains, which display activated MEKK3‐KLF signaling cascade [38]. Indeed, heterozygous endothelial depletion of MEKK3 in the *Ccm2^iECKO^Ccm2l^−/−^* mice (*Ccm2^iECKO^Ccm2l* ^−/−^ *Mekk3^het^*) exhibited potently alleviated CCM lesion burden compared with *Ccm2^iECKO^Ccm2l* ^−/−^ mice. More interestingly, the administration of DPDH was capable of yielding additive effect on ameliorating CCM lesion burden in the *Ccm2^iECKO^Ccm2l* ^−/−^ *Mekk3^het^* mice (Figure S16a–d). Meanwhile, DPDH displayed a similar effect on delaying the development of retinal lesions in the *Ccm2^iECKO^Ccm2l* ^−/−^ *Mekk3^het^* mice as it did in braking the formation of cerebellum lesions (Figure S16e,f). These findings illustrated that DPDH harbored an alternative mechanism in microglial cells for retarding the progression of CCMs in addition to counteracting the MEKK3 signaling cascade in ECs. Ultimately, these results propose that DPDH, as a potent MEKK3 inhibitor, not only blocks the aberrantly activated MEKK3‐KLF2/4 signaling cascade in ECs but also inhibits MEKK3/NF‐κB activation in microglia, thereby attenuating microglial uptake of EC debris and alleviating concomitant inflammatory responses.

To further characterize whether DPDH could play a role in the combination therapy for the treatment of CCMs, we determined the therapeutic synergy of DPDH with celecoxib, which is a clinically used nonsteroidal anti‐inflammatory drug (NSAID) that primarily inhibits COX‐2 activity and has been reported to retard the progression of CCMs [39]. Strikingly, comprehensive assessment through visual inspection, microCT, and retinal vasculature imaging demonstrated that either DPDH or celecoxib treatment alone significantly reduced CCM lesion burden, and their combination potentiated the therapeutic efficacy, yielding substantially greater inhibition on lesion formation and vascular abnormalities than either agent alone (Figure S16g–k). This compelling synergy highlights that co‐targeting MEKK3 signaling and COX‐2 may emerge as a promising therapeutic strategy for CCMs.

### 2.7 DPDH Attenuates Fibrinogen Extravasation and Suppresses Microglial Aggregation Within CCM Lesions

Admittedly, the driving force to recruit the microglial cells to the lesional regions in the *Krit1^ECKO^* mice still remains elusive. Fibrinogen have been described as proteins leaking into the parenchyma upon BBB breakdown. It has been documented that the fibrinogen as a soluble macromolecule leaking into parenchyma upon the disruption of BBB is able to trigger the clustering of perivascular microglial cells, which is essential for the progression of axonal damage in neuroinflammation [40]. In this perspective, we first assessed the roles of fibrinogen in modulating the adhesion and transmigration of HMC3 cells on the sh‐*KRIT1*‐hCMEC/D3 EC monolayer. Intriguingly, it was shown that fibrinogen prominently accelerated the migration of microglial cells towards ECs by virtue of the IBIDI culture‐insert 2 well system (Figure 10a,b). Meanwhile, although 0.5% fibrinogen significantly augmented the migration of microglial cells, there was no obvious difference between 0.5% fibrinogen stimulation and treatment of culture medium collected from the sh‐*KRIT1*‐hCMEC/D3 cells exposed to fibrinogen, implying that fibrinogen plays a decisive role in orchestrating the migration of microglial cells (Figure S17a). Additionally, using a dynamic model of microglial cell adhesion on ECs (shear stress: 2.87 dyns/cm^2^), we found that stimulation of the sh‐*KRIT1*‐HUVECs cells with 0.5% fibrinogen for 2 h contributed to a robust increase in the number of adherent HMC3 microglial cells on the top of sh‐*KRIT1*‐HUVECs cells under flow (Figure 10c,d). Concurrently, immunofluorescence staining of CCM lesions revealed extensive microglial aggregation in the regions with fibrinogen extravasation, which was ameliorated by DPDH through attenuation of fibrinogen leakage (Figures 10e,f and S17b). Moreover, it was demonstrated that ECs encompassed by microglial cells were activated with boosted expression levels of Von Willebrand factor (VWF) and intercellular adhesion molecule‐1 (ICAM‐1) in the serial sections of *Krit1^iECKO^* mice, thereby creating a permissive microenvironment for activated microglia to engulf EC debris (Figures 10g–k and S17c). In line with the data obtained from histological analysis, qRT‐PCR results unveiled that DPDH was sufficient to diminish the mRNA expression levels of *Icam1* and *Vcam1* in the brain ECs isolated from the *KRIT1^iECKO^* mice (Figure S17d). In summary, these results indicate that fibrinogen facilitates the recruitment of microglial cells and further their adhesion to the endothelial layer in CCM lesions, which can be reverse by DPDH through decreasing the leakage of fibrinogen.

**FIGURE 10:**
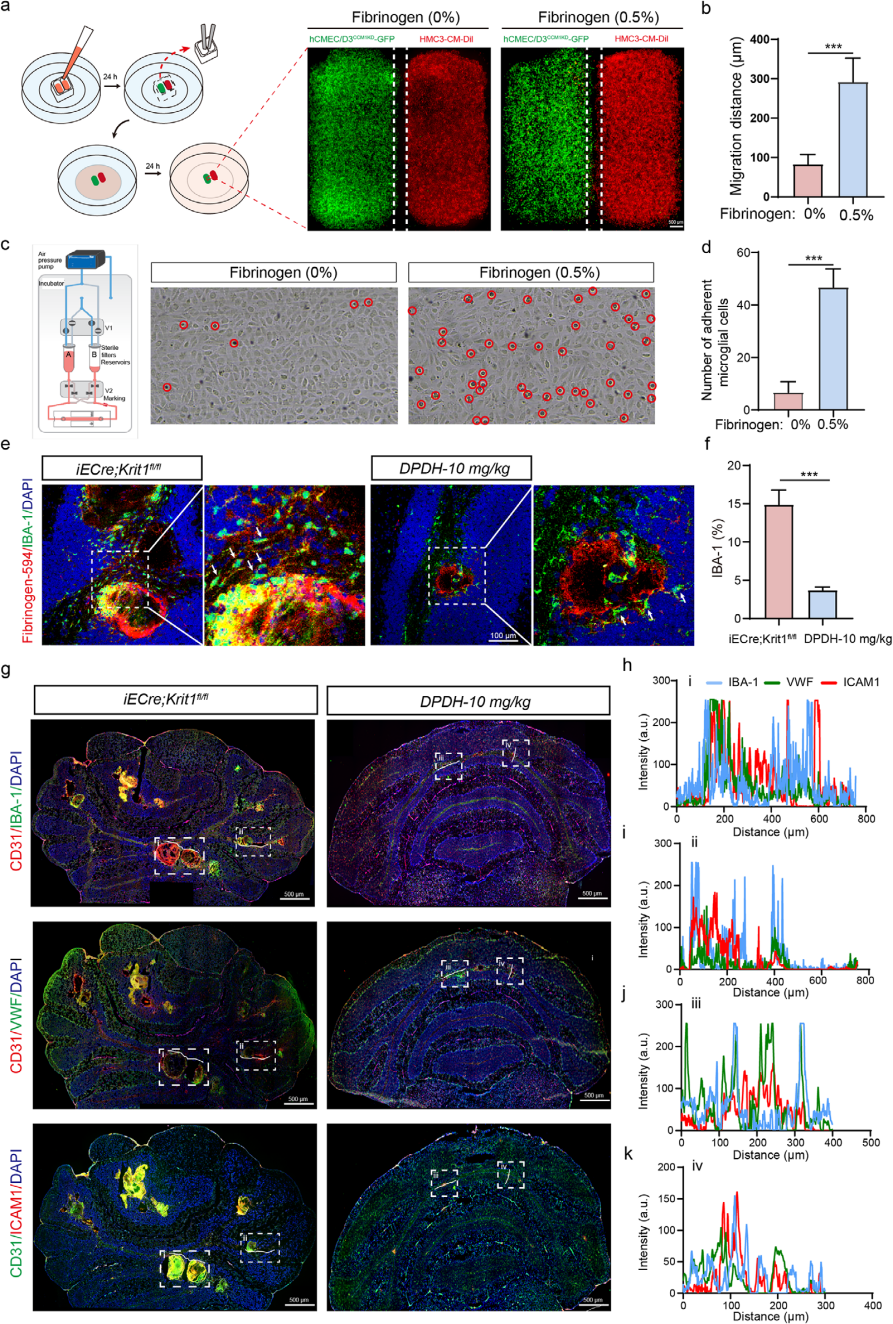
DPDH attenuates fibrinogen extravasation and suppresses microglial aggregation within CCM lesions. a. Left: schematic diagram of migration of microglial cells towards ECs by virtue of IBIDI culture‐insert 2 well system. Right: Representative immunofluorescence staining images of HMC3 microglial cells migrating towards ECs. Scale bar: 500 µm. b. Quantification of the distance of HMC3 microglial cells migrating towards ECs, n = 3. c. Representative images of dynamic adhesion of microglial cells to ECs treated with vehicle control or fibrinogen for 2 h. d. Quantification of the number of adherent microglial cells on top of the ECs treated with vehicle control or fibrinogen, n = 3. e. Representative immunofluorescence images of frozen sections stained with IBA‐1 following the administration of Fibrinogen‐594 in the *Krit1^iECKO^* mice, n = 3. Scale bar: 100 µm. The regions highlighted in white boxes are magnified and the white arrows indicate microglial aggregation in the regions of fibrinogen leakage. f. Quantification of IBA‐1‐positive area (refer to e). g. Representative immunofluorescence staining images for CD31 with IBA‐1, VWF or ICAM1 in the brain sections from *Krit1^iECKO^* mice, n = 3. Scale bar: 500 µm. h‐k. Intensity traces of IBA‐1, VWF, and ICAM‐1 (shown as offset white lines) are plotted. All data are expressed as mean ± SD. ^***^ *p* <0.001.

## 3 Discussion

It has been accepted that the only existing treatment strategy for patients with symptomatic CCM disease is thought to be neurosurgical resection, whereas a large number of CCM patients appear not to be ideal candidates for surgery as a result of the location of lesions or the presence of multiple lesions [6]. Recent genetic studies have demonstrated that activated MEKK3 signaling plays a pivotal role in exacerbating the progression of CCM lesions. Ponatinib, initially identified as a potent tyrosine kinase inhibitor to fight against the development of chronic myeloid leukemia, can strikingly attenuate the lesion burden of CCMs through antagonizing the hyperactive endothelial MEKK3 signaling [41]. However, Ponatinib has been reported to provoke conspicuous adverse effects in the cardiovascular system, but the specific cellular and molecular targets leading to these detrimental effects remain unknown [42]. A kinase inhibition spectrum assay has revealed that ponatinib can target a dozen kinases with high efficiency including MEKK2 [43]. These findings suggest that seeking more specific MEKK3 inhibitors tends to serve as a reliable therapeutic approach to delay the development of CCM disease.

In this study, we illustrate that heterozygous deletion of endothelial MEKK3 is capable of preventing the formation of CCM lesions in the *Krit1^iECKO^* mice at the animal facilities of Nanjing University of Chinese Medicine, validating that MEKK3 acts as an effective and efficient target for treating CCMs. Therefore, identifying potent MEKK3 inhibitors from natural products may represent a promising avenue to seek therapeutic strategies for CCM patients. Currently, AI is increasingly being employed in drug discovery owing to its substantial capacity to reduce timelines and enhance efficacy. Indeed, a plethora of deep learning algorithms have been developed to pave the way for performing novel drug design and target prediction. For example, Lin et al. developed a multimodal AI‐driven Traditional Chinese Medicine (TCM) symptom prediction model (TCM‐SPred) to infer herb–symptom associations, which successfully identified dehydrocostus lactone as a potent renal fibrosis attenuator targeting IQGAP1, showcasing the power of multimodal AI in translating symptom‐based traditional knowledge into mechanistic therapeutic hypotheses [44]. Complementing this knowledge‐driven discovery paradigm, our study leverages AI‐driven *de novo* design to generate a series of small‐molecule compounds as prospective MEKK3 kinase inhibitors. This generative approach enables the exploration of vast chemical spaces beyond existing natural and synthetic compound libraries, allowing for the rapid creation of novel molecular scaffolds tailored to MEKK3. Integrating UNITY 2D fingerprint similarity analysis with in vitro kinase assay data, we found that DHT, among 332 small‐molecule compounds from the traditional Chinese medicine library, most potently reversed elevated MEK5 phosphorylation. Subsequently, we modified the chemical structure of DHT to boost its capability to inhibit the kinase activity of MEKK3, contributing to the synthesis of three new compounds (DPFD, DDNP and DPDH). Notably, 10 µM DPDH markedly inhibited MEKK3 kinase activity with greater potency than DHT, DPFD, and DDNP. Consistently, SPR analysis demonstrated that DPDH bound MEKK3 with the highest affinity. In the present study, in combination with the virtual docking data as well as molecular structure analysis, we posited that the NH group in the imidazole ring of DPDH appeared to be the master functional group for binding to MEKK3 protein. More importantly, a variety of experiments revealed that DPDH tended to directly bind to the Asp44 residue of PB1 domain at MEKK3 protein to prohibit its kinase activity. However, the direct interactions between NH group of DPDH and Asp44 residue of MEKK3 require further validation. Kinome‐wide screening revealed DPDH potently inhibited MEKK3 (99.3% at 10 µM), it also exhibited appreciable inhibition against several other kinases, including HIPK4 (∼64%), JNK2 (∼50%), MSK1 (∼40%), and MEKK2 (∼33%). These data establish MEKK3 as the most sensitive target within the screened panel; however, they do not preclude off‐target activity or permit calculation of IC50 values and selectivity indices. DPDH may inhibit additional kinases at therapeutic concentrations, and that comprehensive IC50‐based selectivity profiling is a critical next step for future drug development.

A series of in vivo assays, including stereomicroscopic brain imaging, microCT, and H&E staining, uncovered that DPDH attenuated CCM progression. Notably, a single DPDH injection yielded 2.4‐fold higher drug concentrations in the cerebellum than in the cerebrum, suggesting preferential accumulation at sites of BBB disruption caused by CCM lesions. During early treatment, compromised endothelial junctions and increased vascular permeability likely facilitated enhanced DPDH extravasation into lesional tissues. However, as DPDH gradually restored BBB integrity and vascular barrier function—as supported by our findings of reduced vascular leakage and tightened endothelial junctions—cerebellar drug penetration might decline over time as the BBB normalized. Therefore, the pharmacodynamic interplay between DPDH distribution and lesion regression appears to be dynamic rather than static. Future investigations should assess time‐course pharmacokinetics to ascertain whether sustained efficacy requires dose adjustment or alternative dosing strategies upon BBB restoration.

It has been well established that disruption in vascular structure and function is a hallmark of CCMs. In fact, brain blood vessels in the lesions of *Krit1^iECKO^* mice are characterized by loosen vascular AJs and TJs, poor pericyte coverage, and compensatory thickening of basement membrane compared to the normal brain blood vessels in the *Krit1^fl/fl^* mice, indicative of damage of vascular structure and function. These phenomena are also observed in the CCM patient samples. Following the treatment of DPDH, the vascular structure in the *Krit1^iECKO^* mice was fortified, with enhanced AJs and TJs, augmented pericyte coverage, as well as normalized basement membrane support. Meanwhile, DPDH improved vascular integrity with strengthened EC junctions and flattened morphology, representing the presence of quiescent endothelium. Notably, a tortuous and disorganized vascular phenotype is visualized in CCM lesions, which is aggravated by the featured shape of mulberry‐like blood vessels. The pathological dilations of blood vessels seen in CCM lesions have preference to yield turbulent, low‐blood flow that precipitates endothelial dysfunction, inflammation and eventually ruptures. Herein, we unveiled that DPDH could reinforce blood flow velocity within the lesions by virtue of functional ultrasound imaging. Moreover, it was shown that DPDH improved vascular function with reduced leakage of EBD and Dextran. Further, transparent processing followed by two‐photon imaging elucidated that DPDH mitigated the enlarged lumen diameter of blood vessels in the CCM cerebellar tissues. These findings imply that DPDH strikingly enhances both the structure and function of cerebral blood vessels in the *Krit1^iECKO^* mice. Mechanistically, activation of the KRIT1‐MEKK3‐MEK5‐ERK5‐KLF2/4 signaling pathway in brain ECs gave rise to the pathological progression of CCMs. DPDH improved endothelial integrity via restricting the activation of MEKK3‐MEK5‐KLF2/4‐pMLC signaling axis. Of interest, S‐flow‐stimulated activation of KRIT1‐MEKK3‐MEK5‐ERK5‐MEF2‐KLF2/4 signaling cascade exerts an atheroprotective effect on adult arterial ECs [45]. As such, examining the differences in ECs during development versus adulthood, in arterial versus in brain capillary, or under distinct hemodynamic conditions can provide implications for revealing the divergent physiological or pathophysiological roles of the activation of KRIT1‐MEKK3‐MEK5‐ERK5‐MEF2‐KLF2/4 signaling pathway [45].

Recent studies have revealed that *Ccm2^iECKO^Ccm2l* ^−/−^ mice displayed more severe CCM phenotypes compared to *Ccm2^iECKO^* mice, and sole inhibition of endothelial MEKK3 can to some degree rescue the CCM phenotypes [38]. To our surprise, it was further illustrated that DPDH led to additive effects on alleviating the lesion burden of CCMs in *Ccm2^iECKO^Ccm2l* ^−/−^ mice upon the heterozygous deletion of endothelial MEKK3, which suggested that DPDH was inclined to take advantage of more complicated mechanisms to retard the progression of CCMs in addition to repressing the MEKK3 signaling in ECs. RNA‐seq of ECs isolated from the *Krit1^iECKO^* mice deciphered that the amelioration of CCM phenotypes in response to DPDH might also rely on the modulation of immune cells. Indeed, microglial cells are deemed to be active surveyors of brain parenchyma with essential functions on sculpting and coordinating neural circuits that respond rapidly to generate a plethora of reactive phenotypes upon infection and damage of brains [19, 46]. Activated microglial cells play vital roles in a panel of acute and neurodegenerative diseases, where they are able to help with clearing neuronal damage through phagocytosis, but can also render progression of brain diseases via secreting multiple components that can trigger a neuroinflammatory state. In this study, PLX5622‐mediated microglial depletion potently prevented the progression of CCMs, suggesting that microglial activation serves as a critical driver of CCM pathogenesis. Furthermore, aberrant phagocytosis of ECs by microglia was detected in clinical CCM specimens, murine CCM models, and in vitro co‐culture systems. Importantly, DPDH effectively suppressed microglia‐mediated phagocytosis of ECs.

In fact, MEKK3 has been gaining increasing attention, in part due to its crucial role in operating on vascular development and further pathological development of CCMs. Knockout of MEKK3 in the process of embryonic or early postnatal development is closely correlated to gross vascular abnormalities [47]. Recently, it has been shown that endothelial‐specific depletion of MEKK3 in adult mice leads to a prominent inward remodeling of both pulmonary and systematic blood vessels, facilitating the occurrence of primary pulmonary and systemic arterial hypertension [29]. Of interest, it has been documented that MEKK3 acts as an important and specific modulator to produce the release of the pro‐inflammatory components including IL‐6 and GM‐CSF rather than the secretion of TNF‐α and IL‐1β in macrophages [22]. Nonetheless, the functions of MEKK3 on microglial cells that are imperative for augmenting inflammation in brains have not been well studied. Herein, we demonstrated that microglial‐specific silence of MEKK3 significantly limited CCM progression. Moreover, DPDH exerted striking effects on restricting microglial uptake of damaged endothelial debris through suppressing the MEKK3‐NF‐κB signaling axis. Notably, all four sporadic CCM clinical specimens exhibited a convergent pathological phenotype characterized by disrupted endothelial barrier integrity, microglial engulfment of damaged endothelial debris, and microglial expression of IL‐6 and IL‐1β, despite quantitative inter‐specimen variation. Sporadic CCMs commonly harbor somatic mutations in *CCM1* (*KRIT1*), *CCM2*, or *CCM3* (*PDCD10*), typically via biallelic inactivation; more recently, somatic mutations in *MAP3K3* and *PIK3CA* have also been identified as frequent contributors to these lesions [48, 49]. Such genetic diversity likely accounts for the quantitative differences observed across our specimens. This phenotypic convergence across genetically distinct lesions reinforces the translational relevance of our findings, suggesting that endothelial barrier disruption coupled with microglial‐mediated clearance of endothelial fragments constitutes a common downstream effector. Nevertheless, our human data derive from a small cohort of sporadic cases with uncharacterized somatic mutation profiles, and that future studies incorporating targeted sequencing of lesion tissue will be necessary to correlate specific somatic genotypes with morphometric phenotypes.

Subsequently, we explored the driving force that microglial cells were recruited to the blood vessels at lesions. It was revealed that, upon permeabilization of vessel wall provoked by the damage of endothelial barrier, various types of components in the blood are prone to diffuse into the brain parenchyma. Serum components including fibrinogen have been regarded as predominant stimulators of microglial cells [50]. Aiming at investigating whether the components derived from serum harbor the capability to steer the recruitment of resident microglial cells, we employed purified fibrinogen to stimulate the HMC3 microglial cells. It was observed that fibrinogen was capable of recruiting microglial cells and further accelerating their adhesion and transmigration in the EC monolayer.

## 4 Conclusion

In conclusion, aberrant MEKK3 signaling in ECs and microglial cells drives the progression of CCMs. We developed a small‐molecule compound named DPDH to serve as a novel MEKK3 inhibitor on the basis of virtual screening, chemical modifications and experimental validation. It was demonstrated that DPDH could delay the development of CCMs through improving vascular structure and preserving BBB function. The underlying mechanisms of DPDH in alleviating CCM lesions mainly include two aspects: firstly, DPDH repressed the aberrant activation of the MEKK3‐MEK5‐KLF2/4‐pMLC2 signaling cascade in ECs, thereby fortifying vascular structure as well as mitigating the leakage of fibrinogen; secondly, DPDH attenuated microglial activation by targeting the MEKK3‐NF‐κB signaling axis within mature lesions, thereby diminishing microglial uptake of damaged EC debris and alleviating local inflammation. Taken together, our study provides a preclinical basis that DPDH emerge as a potential compound to inhibit MEKK3 kinase activity and further exerts the striking effects on preventing the progression of CCMs (Figure 11).

**FIGURE 11:**
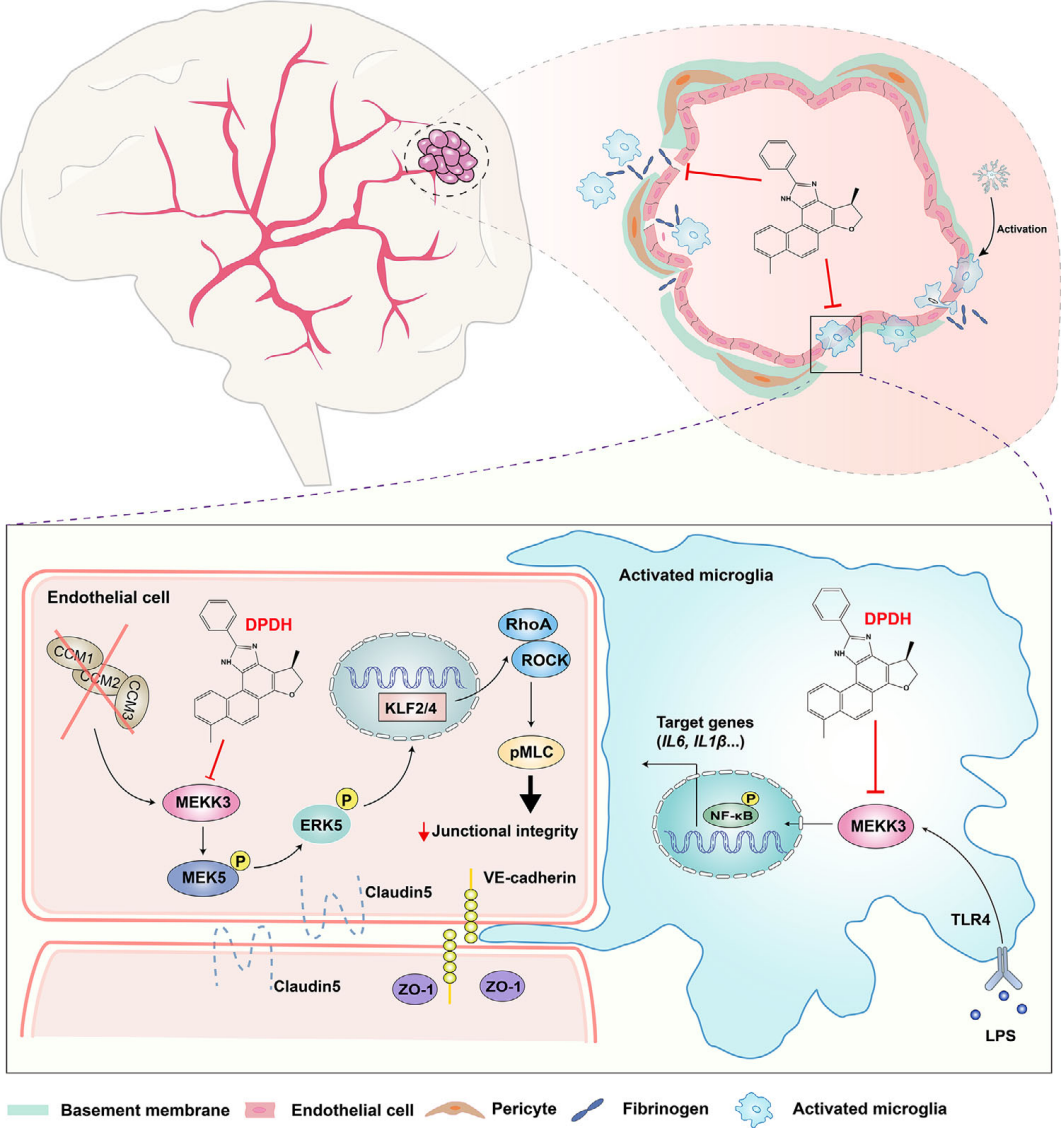
Schematic illustration of DPDH fighting against the progression of CCMs via enhancing endothelial junctional integrity and suppressing microglial activation.

## 5 Materials and methods

### 5.1 Patient Specimens

CCM clinical samples were obtained from Sun Yat‐Sen University Cancer Center. Ethical assessment for the clinical specimens of CCM patients was reviewed and approved by the Medical Ethical Committee of Sun Yat‐Sen University Cancer Center (permit and approval number: B2024‐290), and informed consent was obtained from all participants. All CCM clinical samples were evaluated independently by two pathologists, who were experienced in evaluating immunohistochemistry (IHC) and had no prior information regarding the clinical outcome of the patients. The four collected CCM clinical samples harbored typical CCM lesions with normal surrounding tissues on the basis of hematoxylin and eosin (H&E) staining, and they were further used for immunofluorescent staining. Patient information pertaining to the human CCM samples studied is summarized below.

Sporadic 1: male, age 17, solitary sporadic lesion, removed due to repeated disorders of consciousness and twitching of limbs.

Sporadic 2: female, age 67, solitary sporadic lesion, removed due to repeated dizziness and weakness in limbs accompanied with unsteady walking.

Sporadic 3: male, age 26, solitary sporadic lesion, removed due to repeated headaches and lesion growth.

Sporadic 4: male, age 34, solitary sporadic lesion, removed due to episodic disorders of consciousness.

### 5.2 Mouse Models

All mice were bred on a C57BL/6 background. The *Cdh5*‐*CreERT2* PAC transgenic mice (iECre), *Krit1^fl/fl^*, *Ccm2^fl/fl^*, *Ccm2l* ^−/−^, and *Mekk3^fl/fl^* animals were kindly provided by Jaesung P. Choi (Centre for Inflammation, Centenary Institute, Sydney, Australia) and the generation of the mouse strains used in this study was described previously [38]. All animals were maintained within specific pathogen‐free (SPF) facilities at the Laboratory Animal Center of Nanjing University of Chinese Medicine. All animal protocols were approved by the Animal Care and Use Committee of Nanjing University of Chinese Medicine (approval number: 202208A034 and 202205A024) and performed in accordance with the institutional guidelines of Nanjing University of Chinese Medicine.

### 5.3 Induction of the Neonatal CCM Mouse Models and Treatment Regimens

To induce the endothelial‐specific deletions of *CCM* genes, 50 µL of 4‐hydroxytamoxifen (0.5 mg/mL, H7904, Sigma‐Aldrich) was administered intragastrically to the neonatal pups at P1 [38]. In light of the treatment regimens, vehicle control, dihydrotanshinone‐I (DHT, 10 mg/kg) or DPDH (5 mg/kg or 10 mg/kg) was administrated to the *Krit1^iECKO^ or Ccm2^iECKO^* mice via intraperitoneal injection at P11, P13, P15 and P17. 10 mg/kg of DPDH was intraperitoneally injected into the *Ccm2^iECKO^Ccm2l^−/−^Mekk3^het^* mice (heterozygous deletion of endothelial MEKK3 in the *Ccm2^iECKO^Ccm2l^−/−^* mice) at P7, P9, P11 and P13.

### 5.4 Cell Culture

Human cortical microvascular endothelial cells/D3 (hCMEC/D3; Procell, CL‐0843, China; RRID: CVCL_U985) and human microglial clone 3 (HMC3; Procell, CL‐0620, China; RRID: CVCL_II76) were purchased from Procell Life Science & Technology (Wuhan, China). hCMEC/D3 cells were cultured in Dulbecco's modified Eagle medium (DMEM) supplemented with 10% fetal bovine serum (FBS) and 1% penicillin–streptomycin. HMC3 cells were cultured in Minimum Essential Medium (MEM) supplemented with 10% FBS and 1% penicillin–streptomycin. All cells were maintained at 37°C in a humidified atmosphere containing 5% CO_2_. Human umbilical vein endothelial cells (HUVECs) were isolated and purified from the donated umbilical cords as previously described with minor modification [51], as approved by the Medical Ethical Committee of Jiangsu Province Hospital on Integration of Chinese and Western Medicine (permit and approval number: 2021‐LWKY‐003). All cells used in this study were negative for mycoplasma as periodically tested by a mycoplasma detection kit.

### 5.5 Lentiviral Transduction

The (GFP‐Puro)/U6‐KRIT1‐i and (Cas9‐Puro)/U6‐MEKK3‐KO lentivirus were purchased from Corues Biotechnology (Nanjing, China). HUVECs and hCMEC/D3 were transduced with the (GFP‐Puro)/U6‐ KRIT1‐i lentivirus in the corresponding medium containing 2% (v/v) FBS for 12 h in the presence of HitransG P infection enhancement solution (GeneChem, REVG005, Shanghai, China), after which the medium was removed and replaced by the fresh medium. Following 48 h of transduction, the HUVECs and hCMEC/D3 were collected, and the transfection efficiency was then assessed using the quantitative real‐time reverse‐transcription PCR (qRT‐PCR) assay. HMC3 cells were transduced with the (Cas9‐Puro)/U6‐MAP3K3‐KO lentivirus for 12 h, followed by that the medium was removed and replaced with fresh medium. The cells were collected and used for subsequent experiments 72 h after transduction. The sequences of gene‐specific primer sets used in this study are provided in Table S1.

### 5.6 Generative Schemes for De Novo Drug Design With AI

The generative schemes for *de novo* drug design with AI were performed as previously described [52, 53]. In brief, the “simplified molecular‐input line‐entry system (SMILES)” structures of small‐molecule compounds culled from ChEMBL database were utilized to train the “Recurrent Neural Network (RNN)” for obtaining the syntax of SMILES, and the RNN was able to originate the small‐molecule compounds by virtue of representation from the conditional probability distribution related to the training group. Subsequently, a deep learning model that integrates recurrent and convolutional neural networks was proposed to predict compound–protein affinity. The small‐molecule compound with predicted lowest half‐maximal inhibitory concentration (IC50) value or highest pIC50 value was selected for performing subsequent structure similarity comparison.

### 5.7 In Vitro Kinase Assay

For the in vitro kinase assay, the purified glutathione S‐transferase (GST)‐MEK5 recombinant fusion protein was employed as a substrate. The purified FLAG‐MEKK3 and GST‐MEK5 recombinant proteins were incubated in the kinase reaction buffer system [20 mM HEPES/KOH (pH 7.5), 5 mM MgCl_2_, 2 mM dithiothreitol, 1 mM adenosine triphosphate (ATP)] for 4 h at 30°C [41]. The reaction mixtures were subsequently denatured with Laemmli loading buffer, and western blot analysis was performed according to a standard protocol. Phosphorylated MEK5 (p‐MEK5) was detected using the p‐MEK5 antibody (NB100‐61105, Novus Biologicals, CO, USA) to reflect the enzyme activity of MEKK3.

### 5.8 Whole‐Mount Immunofluorescence Staining of Mouse Retina

Whole‐mount immunofluorescence staining of mouse retina was conducted as previously described [54]. Briefly, the eyes were collected from neonatal mice at P14 (*Ccm2^iECKO^Ccm2l^−/−^* mice) or P19 (*Krit1^iECKO^* mice), and then the harvested eyes were fixed in 4% paraformaldehyde (PFA) solution in phosphate‐buffered saline (PBS) for 2 h on ice. In light of dissection of retinas, the cornea, lens, sclera, and hyaloid vessels were carefully removed. The retinas were permeabilized and blocked in PBS containing 0.5% Triton and 5% BSA overnight at 4°C, followed by incubation with indicated primary antibodies diluted 1:100 in PBS containing 0.1% Triton X‐100 and 1% BSA overnight at 4°C. On the following day, the retinas were washed in PBS and then incubated with the corresponding fluorescent secondary antibodies overnight in the dark at 4°C. Subsequently, the retinas were washed five times in PBS, and then flattened and mounted through making four incisions in the fluorescent mounting medium. The images were acquired with the same exposure and gain settings using a two‐photon confocal microscope (Leica STELLARIS 8 DIVE, Heidelberg, Germany) or THUNDER Imager (Leica THUNDER Imaging Systems, Heidelberg, Germany). The images of retinal blood vessels with different immunofluorescence staining were analyzed using ImageJ software (National Institutes of Health, USA).

### 5.9 Microcomputed Tomography (microCT) Scan

X‐ray microCT was utilized to quantify CCM lesions, as previously described with minor modifications [55]. In brief, the neonatal mice were perfused with PBS containing 2% PFA into the ventricle of the mouse heart. After perfusion, the brains were quickly dissected and fixed overnight in 4% PFA. Subsequently, the hindbrains were detached using forceps and washed with PBS solution. After that, the mouse hindbrains were soaked overnight in Lugol's iodine solution (L6146, Sigma‐Aldrich, Germany). Ultimately, the hindbrains were randomized and scanned by a blinded operator using PerkinElmer MicroCT system (PerkinElmer, Quantum GX2). MicroCT analysis for quantification of CCM lesions was performed in accordance with the standard procedure of microCT analysis software.

### 5.10 Functional Ultrasound for High‐Resolution Imaging of Brain Vasculature

High resolution imaging of brain blood vessels and blood flow was performed using an ultrasound localization microscopy (Iconeus One, Paris, France), as previously described with minor modifications [56]. Briefly, the mice were anaesthetized using isoflurane, followed by the moue head was placed in a stereotaxic frame. Subsequently, the mouse scalp was removed with scissors to expose the intact skull. Following surgery, the brain was gently rinsed with sterile saline, and then ultrasound coupling gel was placed on the mouse skull. After retro‐orbital venous sinus injection of ultrasound contrast‐agent consisting of sulphur hexafluoride (SF6) microbubbles (SonoVue) dissolved in 0.9% NaCl into the mice, the ultrasound images were continuously recorded for 8 min. The dedicated imaging sequence for blood flow intensity and velocity as well as the live reconstruction procedure were implemented in a live acquisition software (IcoScan, Iconeus, Paris, France).

### 5.11 Surface Plasmon Resonance (SPR) Analysis

The SPR measurements were carried out using a Biacore T200 instrument (GE Healthcare, Uppsala, Sweden) according to the manufacturer's instructions. Briefly, the human recombinant MEKK3 was diluted to 50 µg/mL with 10 mmol/L sodium acetate (pH 5.0) and then covalently immobilized on a CM5 sensor chip (Series S Sensor Chip, GE Healthcare). Subsequently, the mixture was serially diluted with the running buffer (0.05% Tween 20 dissolved in PBS) and then injected at a flow rate of 30 mL/min. The binding affinity was evaluated with the equilibrium dissociation constant (KD) value, which was acquired through fitting the data to a steady state affinity model using Biacore T200 Evaluation Software.

### 5.12 Isolation and Purification of Mouse Cerebellar ECs

Mouse cerebellar ECs were isolated and purified via enzymatic digestion, followed by separation using magnetic‐activated cell sorting by virtue of anti‐CD31‐conjugated magnetic beads (MACS MS system, Miltenyi Biotec, Germany), as previously described with minor modifications [41]. In brief, the mouse pups at P19 were subjected to isoflurane anesthesia treatments and then perfused with sterile PBS. The cerebellar tissues were prepared into single‐cell suspensions using Adult Brain Dissociation Kit (130‐107‐677, Miltenyi Biotec, Germany). Subsequently, the cells were centrifuged, resuspended, and incubated with anti‐mouse CD31 antibody conjugated microbeads for 15 min at 4°C. The microbead‐bound cells were then washed and separated using MACS MS columns as per the vendor protocol. The cells bound to the magnetic column were eluted and centrifuged for further experiments, including RNA‐sequencing (RNA‐seq) and qRT‐PCR analysis.

### 5.13 RNA‐seq

The TruePrep DNA Library Prep Kit V2 (TD503, Vazyme, China) was utilized to prepare the low‐input library. Next, the 2 × 150 bp paired‐end sequencing (PE150) was conducted on an Illumina Novaseq 6000 (Gene Denovo Technology Co., Ltd., China) according to the manufacturer's protocol. Following the mRNA profiles were generated, StringTie was employed to determine the expression levels of all transcripts by calculating fragments per kilobase of exon model per million mapped reads (FPKM). The differentially expressed mRNAs were selected with |log2(fold change)| >1 and *p* value <0.05 by the R package, and then gene ontology (GO) enrichment analysis was performed on the differentially expressed mRNAs. RNA sequencing data that support the findings of this study have been deposited in NCBI Gene Expression Omnibus (GEO) under accession number GSE303628.

### 5.14 Fluorescein Isothiocyanate (FITC)‐dextran Perfusion, Evan Blue Dye (EBD) and Fibrinogen Leakage Assays

For the FITC‐dextran perfusion assay, FITC‐dextran (FD2000S; Sigma‐Aldrich, USA) was dissolved in 0.9% NaCl at a concentration of 50 mg/ml, followed by centrifugation at 10,000 × g for 5 min. The supernatants were collected and kept protected from light for subsequent injection. The retro‐orbital venous sinus injection of FITC‐dextran (10 µl per gram body weight) was performed as previously described [25]. 5 min following the injection, the mouse brains were collected and fixed for further sectional or whole‐mount immunofluorescence staining. In light of EBD or Alexa594‐fibrinogen leakage assay, EBD (100 µl of a 1% solution in 0.9% NaCl) or Alexa594‐fibrinogen (5 g/ml, 10 µl per gram body weight) was injected into the retro‐orbital plexus of anesthetized mouse pups at P19. 30 min following the injection, the mouse pups were sacrificed and perfused with PBS via the left ventricle to get rid of the dye from the vascular volume. The cerebellum tissues and retinas of mouse pups were collected and fixed for subsequent whole‐mount immunofluorescence staining.

### 5.15 Two‐Photon Microscopy Imaging

In order to visualize the leakage of blood vessels in the cerebellar tissues in a three‐dimensional (3D) manner, two‐photon microscopy imaging was utilized. Briefly, EBD or FITC‐dextran was injected into the retro‐orbital plexus of anesthetized mouse pups at P19. Next, the tissue transparency reagents (T3703 and T3741, TCIchemicals, China) were utilized to treat the harvested cerebellar tissues. Subsequently, the treated samples were imaged under the 25× water immersion objective of two‐photon confocal microscopy (STELLARIS 8 DIVE, Leica, Heidelberg, Germany).

### 5.16 Microglial Cell Adhesion Assay

In vitro adhesion assay of microglial cells onto ECs under dynamic flow was carried out as previously described with minor modifications [57]. In brief, HUVECs were seeded onto the µ‐Slide VI 0.5 Glass Bottom (Ibidi, Planegg, Germany) and allowed to grow confluence. HUVEC monolayers were subsequently stimulated with 0.5% fibrinogen for 2 h prior to the treatment of shear stress. The microglial cells in HUVEC medium at the density of 10^6^ cells/mL were placed into the ibidi Pump System (Ibidi, Planegg, Germany). The dynamic adhesion of microglial cells onto HUVECs treated with vehicle control or 0.5% fibrinogen was recorded (2.87 dyn/cm^2^ constant shear stress) with an inverted phase‐contrast microscope. The number of adherent microglial cells onto HUVECs per field were quantified using ImageJ software (National Institutes of Health, USA).

### 5.17 In Vitro Debris Phagocytosis Assay

Death of ECs was induced by repeated freeze‒thaw cycles (3 cycles) as previously described [58]. Dead ECs were labeled with CFSE at 37°C for 30 min and then washed 3–4 times with PBS. To assess whether cell debris could be engulfed by microglia, CFSE‐labeled debris was added to the cell cultures at a debris‐to‐cell ratio of 10:1. Non‐engulfed debris was thoroughly washed away with PBS, and the engulfment of debris was subsequently analyzed using a THUNDER Imager (Leica THUNDER Imaging Systems, Heidelberg, Germany).

### 5.18 Adeno‐Associated Virus Injection

All AAV expression vectors were constructed, packaged and molecularly verified by Corues Biotechnology (Nanjing, China). The shRNA AAV vector targeting MEKK3 in microglia was used for in vivo knockdown. The designed AAV plasmids for MEKK3 shRNA and scramble were constructed as AAVphp.eb‐Iba1‐Cre‐shMEKK3 and AAVphp.eb‐Iba1‐Cre‐shScram, respectively. P5 littermates were administered 10 µl of PBS containing 2 × 10^10^ (2 × 10^10^ genome copies [GC] AAVphp.eb‐Iba1‐Cre‐shScram, 2 × 10^10^ GC AAVphp.eb‐Iba1‐Cre‐shMEKK3) GC/mouse of AAV by retro‐orbital venous sinus injection as described previously [59].

### 5.19 Statistical Analysis

All data are presented as mean ± standard deviation (SD). If the *p* value was less than 0.05, the results were considered statistically significant, otherwise indicated as nonsignificant (ns). Data determined to be parametric were analyzed using an unpaired Student's t‐test (comparing the means of two groups), or ordinary one‐way analysis of variance (ANOVA) (comparing differences of means among more than 2 groups). All the graphs, calculations and statistical analyses were performed using GraphPad Prism software version 8.0 (San Diego, USA).

## Author Contributions

**Conceptualization: Yang Zhao**, **Yin Lu**, **Suyun Yu**, **Jia Li**; **Methodology: Weiwei Zheng**, Suyun Yu, **Liwenyu Chen**, **Jing Ma**, **Xiaoqing Xin**, **Qiuhong Shen**, **Jing Chen**, **Song Ke**, **Hao Liu**, **Ruogu Qi**; **Software**: Weiwei Zheng, Liwenyu Chen, Song Ke, Hao Liu; **Data curation**: Weiwei Zheng, **Chang Yu**; **Investigation**: Weiwei Zheng, Liwenyu Chen, **Peiliang Shen**, Xiaoqing Xin; **Validation**: Weiwei Zheng, Jing Ma, Peiliang Shen, Qiuhong Shen; **Formal analysis**: Suyun Yu, Liwenyu Chen; Supervision:Suyun Yu, Jing Chen, Jia Li, Yin Lu, Yang Zhao; **Funding acquisition**: Weiwei Zheng, Yang Zhao; **Visualization**: Weiwei Zheng, Peiliang Shen, Chang Yu; **Project administration**: Weiwei Zheng, Yang Zhao; **Resources**: Xiaoqing Xin, Jing Chen, Song Ke, Hao Liu, **Xiaobing Jiang**, Ruogu Qi, **Juming Yan**, Jia Li, **Jaesung P. Choi**, Yin Lu; **Writing – original draft**: Weiwei Zheng; **Writing – review & editing**: Suyun Yu, Yin Lu, Yang Zhao.

## Funding

This work was financially supported by the projects of National Natural Science Foundation of China (82274233, 82574731, 82608254), the Natural Science Foundation of Jiangsu Province (BK20240146), the Science and Technology Development Fund (Macau SAR, 0003/2025/NRP), Innovation Project of Jiangsu Key Laboratory for Pharmacology and Safety Research of Chinese Materia Medica (2026JKLPSRCMM12), Innovation Project of SKL of Technologies for Chinese Medicine Pharmaceutical Process Control and Intelligent Manufacture (NZYSKL2601), Key Program of Natural Science Foundation for Jiangsu Higher Education Institutions (22KJA360007), the National Key Research and Development Program of China (2025YFC3507500), Noncommunicable Chromic Diseases‐ National Science and Technology Major Project (2024ZD0530800), the China Postdoctoral Science Foundation (2025M783888) and the Postdoctoral Fellowship Program (Grade C) of China Postdoctoral Science Foundation (GZC20262146).

## Conflicts of Interest

The authors declare no conflicts of interest.

## Supporting information




**Supporting File 1**: advs78132‐sup‐0001‐SuppMat.docx.

**Supporting File 2**: advs78132‐sup‐0002‐SuppMat.docx.

**Supporting File 3**: advs78132‐sup‐0003‐TableS2.xlsx.

**Supporting File 4**: advs78132‐sup‐0004‐MovieS1.mp4.

**Supporting File 5**: advs78132‐sup‐0005‐MovieS2.avi.

**Supporting File 6**: advs78132‐sup‐0006‐MovieS3.avi.

**Supporting File 7**: advs78132‐sup‐0007‐MovieS4.avi.

**Supporting File 8**: advs78132‐sup‐0008‐MovieS5.avi.

**Supporting File 9**: advs78132‐sup‐0009‐MovieS6.avi.

**Supporting File 10**: advs78132‐sup‐0010‐MovieS7.avi.

**Supporting File 11**: advs78132‐sup‐0011‐MovieS8.avi.

**Supporting File 12**: advs78132‐sup‐0012‐MovieS9.avi.

**Supporting File 13**: advs78132‐sup‐0013‐MovieS10.avi.

## Data Availability

The data that support the findings of this study are available on request from the corresponding author. The data are not publicly available due to privacy or ethical restrictions.
