# Supplementary material for: Inhibition of MEKK3 Prevents Cerebral Cavernous Malformation Progression Via Restoring Endothelial Integrity and Restricting Microglial Activation
Source: Adv Sci (Weinh). 2026 Oct 6:e78132. Online ahead of print. doi: 10.1002/advs.78132 (PMC13643112; doi:10.1002/advs.78132)
Supplement: Supplementary file 1 [file ADVS-9999-e78132-s003.docx]

**Supplementary Information**

**Inhibition of MEKK3 prevents cerebral cavernous malformation progression via restoring endothelial integrity and restricting microglial activation**

Weiwei Zheng^1,2,†^, Suyun Yu^1,2,†^, Liwenyu Chen^1,2^ , Jing Ma^1,2^, Peiliang Shen^2^, Chang Yu^1,2^, Xiaoqing Xin^2^, Qiuhong Shen^2^, Jing Chen^2^, Song Ke^3^, Hao Liu^3^, Xiaobing Jiang^4^, Ruogu Qi^2^, Juming Yan^5^, Jia Li^6^, Jaesung P. Choi^7^, Yin Lu^1,8,*^, Yang Zhao^1,2,*^

*^1^Jiangsu Key Laboratory for Pharmacology and Safety Research of Chinese Materia Medica, Nanjing University of Chinese Medicine, Nanjing, China*

*^2^Department of Biochemistry and Molecular Biology,* *School of Medicine, Nanjing University of Chinese Medicine, Nanjing, China*

*^3^Matwings Technology Company, Shanghai, China*

*^4^State Key Laboratory of Oncology in South China, Collaborative Innovation Center for Cancer Medicine, Department of Neurosurgery, Sun Yat-sen University Cancer Center, Guangzhou, China*

*^5^Jiangsu Key Laboratory of Immunity and Metabolism, Department of Pathogenic Biology and Immunology, National Experimental Demonstration Center for Basic Medicine Education, Xuzhou Laboratory of Infection and Immunity, Xuzhou Medical University, Xuzhou, China*

*^6^Curtin Medical Research Institute and Curtin Medical School, Curtin University, Bentley, WA, Australia*

*^7^**School of Biomedical Sciences, University of New South Wales, Sydney, NSW, Australia*

*^8^State Key Laboratory of Mechanism and Quality of Chinese Medicine, Macau University of Science and Technology, Taipa, Macau SAR, China*

^*^Corresponding authors.

E-mail addresses: y.zhao@njucm.edu.cn (Yang Zhao) and luyingreen@njucm.edu.cn (Yin Lu).

^†^These authors contributed equally to this work.

1. **MATERIALS AND METHODS：**

**1.1 Reagents and antibodies**

The reagents used in this study are listed as follows: Nobiletin (HY-N0155, MCE, USA), Tangeretin (HY-N0133, MCE, USA), Pseudocoptisine chloride (HY-N6894A, MCE, USA), Corydaline (HY-N0923, MCE, USA), Osthol (HY-N0054, MCE, USA), Oroxindin (HY-N0399, MCE, USA), Physcion (HY-N0108, MCE, USA), Formononetin (HY-N0183, MCE, USA), Dihydrotanshinone I (T92895, Yuanye, China), Luteolin (HY-N0162, MCE, USA), Benzaldehyde (B802738, Macklin, China), Ammonium acetate (A800996, Macklin, China), o-Phenylenediamine (P815627, Macklin, China), 2,3-Dichloro-5,6-dicyano-p-benzoquinone (D807305, Macklin, China), Ethanol (E809061, Macklin, China), Celecoxib (HY-14398, MCE, USA), Latex beads (L2778, Sigma, USA). The antibodies used in this study are listed as follows: MEK5 (40737S, CST, USA), p-MEK5 (NB100-61105, Novus, USA), MEKK3 (5727S, CST, USA), CD31 (557355, BD, USA), CD31 (3528S, CST, USA), Isolectin B4 (B-1205-.5, Vector laboratories, USA), ZO-1 (13663S, CST, USA), VE-Cad (2500, CST, USA), Claudin5 (49564S, CST, USA), NG2 (ab259324, Abcam, UK), Collagen IV (ab236640, Abcam, UK), KLF2 (ab194486, Abcam, UK), KLF4 (AF3158-SP, RD, USA), ERK5 (12950S, CST, USA), p-ERK5 (3371S, CST, USA), ERK1/2 (AF0155, Affinity, Australia), p-ERK1/2 (RA2202, Vazyme, China), NF-κB p65 (8242, CST, USA), p-NF-κB p65 (3033, CST, USA), P38 (8690, CST, USA), p-P38 (4511, CST, USA), JNK (9252, CST, USA), p-JNK (4688, CST, USA), Fibrinogen alpha chain (69708, CST, USA), CD41 (133903, Biolegend, USA), CD68 (137002, Biolegend, USA), IBA-1 (17198, CST, USA), VWF (ab287962, Abcam, UK), ICAM1 (sc-71292, Santa Cruz Biotechnology, USA), VCAM1 (DF6082, Affinity, Australia), IL-6 (12912, CST, USA), IL-1β (12242, CST, USA), PAD4 (ab214810, Abcam, UK), citH3 (ab5103, Abcam, UK), APC anti-mouse CD11b (101211, Biolegend, USA), Alexa Fluor® 700 anti-mouse CD45 (147715, Biolegend, USA), APC/Cy7 anti-mouse CD45 (103115, Biolegend, USA), FITC anti-mouse CD4 (100405, Biolegend, USA) and PE anti-mouse CD8 (100707, Biolegend, USA), PE anti-mouse CD31 (160203, Biolegend, USA), FITC anti-mouse CX3CR1 (149019, Biolegend, USA), Goat anti-Rabbit IgG (H+L) Cross-Adsorbed Secondary Antibody, Alexa Fluor™ 488 (A11008, Invitrogen, USA), Goat anti-Mouse IgG (H+L) Cross-Adsorbed Secondary Antibody, Alexa Fluor™ 488 (A11001, Invitrogen, USA), Goat anti-Rat IgG (H+L) Cross-Adsorbed Secondary Antibody, Alexa Fluor™ 488 (A11006, Invitrogen, USA), Goat anti-Rabbit IgG (H+L) Cross-Adsorbed Secondary Antibody, Alexa Fluor™ 594 (A11012, Invitrogen, USA), Goat anti-Rat IgG (H+L) Cross-Adsorbed Secondary Antibody, Alexa Fluor™ 647 (A21247, Invitrogen, USA), Peroxidase-conjugated goat anti-mouse IgG (H+L) peroxidase-conjugated goat anti-mouse IgG (H+L) (33201ES60, YEASEN, China), Peroxidase-conjugated goat anti-rabbit IgG (H+L) peroxidase-conjugated goat anti-mouse IgG (H+L) (33101ES60, YEASEN, China).

**1.2 Synthesis of DPFD, DDNP and DPDH**

DPFD formation: DHT (0.1 mmol) in benzene (15 ml) was refluxed overnight with 2,3-dichloro-5,6-dicyano-l,4-benzoquinone (0.5 mmol). After thin-layer chromatography (TLC) monitoring showed the completion of the reaction, the solvent was evaporated and the residue was subjected to Preparative Thin-Layer Chromatography (PTLC) isolation (Pet/EtOAc=2/1).

DDNP formation: DHT (0.1 mmol) in EtOH was added O-phenylenediamine (0.15 mmol) The solution was heated to reflux overnight. After TLC monitoring showed the completion of the reaction, the solvent was evaporated and the residue was subjected to PTLC isolation (Pet/CH_2_Cl_2_=5/1).

DPDH formation: Solution of DHT (0.1 mmol), benzaldehyde (0.2 mmol, 2 eq) and aminoacetate (0.4 mmol, 4 eq) in EtOH (0.1 M) was heated to reflux for 6 hours. After TLC monitoring showed the completion of the reaction, the solvent was evaporated and the residue was subjected to PTLC isolation (Pet/EtOAc=2/1).

**1.3 Western blot**

In brief, the proteins were prepared from the hCMEC/D3 cells or HMC3 cells using the radioimmunoprecipitation assay (RIPA) buffer. The proteins were then separated on the basis of size through gel electrophoresis, followed by that the proteins were transferred onto a polyvinylidene difluoride (PVDF) membrane. After incubation with the indicated primary and secondary antibodies, the protein detection and blot visualization were performed according to the standard protocols. Glyceraldehyde phosphate dehydrogenase (GAPDH) was used as invariant control.

**1.4 Hematoxylin and eosin (****H&E) staining**

Collected mouse brains were fixed in 4% PFA overnight, dehydrated in 100% ethanol, and embedded in paraffin. H&E staining was performed on the 8 μm thick sections according to the manufacture’s protocols. The stained sections were imaged using the Mantra Pathology Workstation (PerkinElmer, Waltham, USA).

**1.5 Drug affinity responsive target stability (DARTS) assay**

Briefly, H293T cells overexpressing MEKK3 were lyzed with M-PER lysis buffer (78503, Thermo Fisher, USA) containing protease (ST506, Beyotime, China) and phosphatase inhibitors (P1082, Beyotime, China). The lysates were subjected to centrifugation at 12,000 rpm for 15 min at 4°C, after which the supernatants were collected. Subsequently, the supernatants were divided into two equal parts, and incubated with either DMSO or DPDH at room temperature for 30 min. Each part of lysates was then equally divided into five fractions, and proteolysis was performed in the absence or presence of different ratios of pronase (10165921001, Roche, Switzerland) to protein for 30 min at room temperature. The reaction was terminated via adding loading buffer and boiling immediately. The resulting samples were loaded onto 10% sodium dodecyl-sulfate polyacrylamide gel electrophoresis (SDS-PAGE) for further western blot analysis.

**1.6 Cellular thermal shift assay (CETSA)**

H293T cells overexpressing MEKK3 were incubated in the absence or presence of DPDH for 2 h, the cells were then collected and subjected to CETSA assay as previously described with minor modifications^2^. In brief, the collected cells were equally divided into 10 parts, and each part of cells was heated for 3 min under different temperature (44, 46, 48, 50, 52, 54, 56, 58, 60, 62°C). Subsequently, the heated cells were frozen at −80°C for 12 h and then incubated at room temperature for 5 min. The cells were then subjected to three freeze-thaw cycles, after which the cell lysates were extracted through centrifugation at 20,000 × g for 20 min. The protein level of MEKK3 was detected by western blot analysis.

**1.7 Micro scale thermophoresis (MST)**

In brief, the purified his-tagged human recombinant MEKK3 (N-6His) protein (PH32110E1, Abicenter, USA) was labeled with RED dyes (MO-L018, NanoTemper Technologies, Germany). An aliquot (10 μL) of the serial concentration dilution of DPDH was mixed with 10 μL of labeled MEKK3 protein. Subsequently, the mixed samples were loaded into the standard capillaries (K002, NanoTemper Technologies, Germany), and the MST analysis was carried out on the Monolith NT.115 MST instruments (NanoTemper Technologies, Germany). The data were analyzed using the MO. Affinity Analysis 3 software.

**1.8 Quantitative real-time reverse-transcription PCR (qRT-PCR)**

Total RNA was extracted from cells using TRIzol reagent (Vazyme, Nanjing, China) according to the manufacturer’s instructions and then reversely transcribed to cDNA with Hifair III Reverse Transcriptase (YEASEN, Shanghai, China). SYBR Green Master Mix (Vazyme, Nanjing, China) was used to perform qPCR on the Applied Biosystems 7500 Real-Time PCR Systems (Thermo Fisher Scientific, USA). GAPDH was used as a reference control, and the mRNA expression levels of genes were represented as relative fold changes normalized to GAPDH controls. The primer sequences used in this study are listed in Supporting Table S1.

**1.9 Flow cytometry analysis**

*Krit1^iECKO^* mice treated with or without DPDH were sacrificed, and then the brains were collected and placed into cold solution A (0.5% BSA in DMEM, 1 μg/μl glucose, 10 mM HEPES, and 1x penicillin-streptomycin). Meninges, brain stem, white matter/brainstem were carefully removed. The brain tissues were triturated using scissors and centrifuged at 1000 g for 5 min. The supernatants were removed, and the pellets were incubated in the pre-warmed collagenase/dispase solution for 40 min at 37℃ (with vigorous shaking every 10 min). Following incubation, cold solution A was added and then the suspension was centrifuged at 600 g for 10 min at 4℃. The supernatants were removed, and the cell pellets were resuspended in a Percoll gradient (70% Percoll, 37% Percoll, 30% Percoll, respectively) followed by centrifugation at 1000 g for 25 min at room temperature. The final solution consisted of three distinct layers, with a buffy leukocyte layer at the interface of clear 70% Percoll and 37% Percoll, and with a thick myelin layer at the top of the tube. The leukocyte layer was extracted and resuspended in cold PBS in a fresh tube, followed by centrifugation at 600 g for 7 min at 4℃. The supernatants were removed, and cells were resuspended in flow cytometry buffer (0.5% BSA, 2 mM EDTA in PBS). After the isolated cells were stained in the dark for 30 min at 4℃ with a mixture of PI, anti-mouse CD11b-APC and Alexa Fluor® 700 anti-mouse CD45 antibodies, or a mixture of anti-mouse CD45-APC-Cy7, CD4-FITC and CD8-PE antibodies. After incubation, the cells were then analyzed on Beckman coulter cell analyzer.

**1.10 Kinome-wide screening of DPDH**

Firstly, 311 potential kinase targets of DPDH were identified through virtual screening using the KIPP database (https://kipp.idruglab.cn). Next, we prioritized 110 kinases closely related to CCM pathogenesis based on compensatory signaling pathways, structural domain homology, and kinase family relationships. Intersection analysis yielded 46 critical kinases, which were further experimentally profiled for determining DPDH sensitivity (Carna Biosciences, Inc).

**1.11 Pharmacokinetic studies**

10 mg/kg of DPDH or DHT was administrated intraperitoneally into the *Ccm1^iECKO^* mice. The cerebrum and cerebellum tissue samples were collected from each animal at the indicated time points and then stored in a freezer at −80°C. All collected samples with the treatment of tested compounds were analyzed by LC−MS/MS.

**1.12 Acute toxicity studies**

C57BL/6J mice (6–8 weeks old) were purchased from Shanghai SLAC Laboratory Animal Co., Ltd. (Shanghai, China). All animals were maintained within specific pathogen-free (SPF) facilities at the Laboratory Animal Center of Nanjing University of Chinese Medicine. The animal protocols of acute toxicity studies were approved by the Animal Care and Use Committee of Nanjing University of Chinese Medicine (approval number: 202505A070) and performed in accordance with the institutional guidelines of Nanjing University of Chinese Medicine. Briefly, seventy C57BL/6J mice were randomly divided into seven groups (each group consisting of 5 males and 5 females). The groups were designated as the control group, vehicle group, and 20, 40, 80, 160, and 320 mg/kg DPDH groups, respectively. Routine observations were made daily for 7 days, after which the major organs (brain, liver, heart, lungs, kidneys, and spleen) were harvested, fixed with 4% PFA, and embedded in paraffin. The 8 μm histological sections were stained with H&E. The images were acquired under the Mantra Pathology Workstation (PerkinElmer, Waltham, USA).

**1.13 Immunofluorescence analysis**

Mouse brains were harvested after the euthanasia of animals, fixed with 4% PFA, embedded in optimal cutting temperature (OCT), and cut to 15 or 30 μm thick sections. The slides were washed in PBS twice to remove OCT and then blocked in 5% goat serum in 0.3% TritonX-100 in PBS for 30 min to prevent non-specific staining and to permeabilize the tissues. Subsequently, the slides were incubated with the indicated primary antibodies against CD31, Claudin-5, Collagen-IV, NG2, VE-cadherin, ZO-1, IBA1, CD68, CD41, IB4 or fibrinogen alpha chain overnight at 4°C. Next, the slides were washed in PBS three times and then incubated with corresponding secondary antibodies at room temperature for 1 h. Ultimately, the images were taken using a Thunder microscope (Thunder, Leica, Germany).

**1.14 Scanning electron microscope (SEM)**

Mice were perfused with 10 ml of SEM fixative [2.5% electron microscopy grade glutaraldehyde, 2% formaldehyde, 2 mM calcium chloride, 2% sucrose, and 0.1 M cacodylate buffer (pH 7.4)]. Subsequently, the hindbrains were dissected and fixed in the SEM fixative overnight. The morphology of blood vessels in hindbrains was examined using a SEM (HITACHI U8010, Tokyo, Japan).

**1.15 Laser Speckle Contrast Imaging**

The cerebral blood flow (CBF) was evaluated by using a laser speckle contrast imaging system (RWD Life Sciences, China). In brief, the mouse scalp was shaved and sterilized with povidone-iodine and 70% ethanol, and then was incised to expose the skull. The skull surface was cleaned and moistened with sterile saline. Real-time changes of CBF were recorded with a CCD camera. The same region of interest (ROI) was defined for all groups. The images of CBF were analyzed using a RFLSI analysis software (RWD Life Sciences, China).

**1.16 Depletion of microglia**

To establish microglia-free niches, the *Krit1^iECKO^* mice were treated with PLX5622, a CSF1R inhibitor. Specifically, P11 mice were intraperitoneally injected at a dosage of 50 mg per kg of mouse body weight for 7 consecutive days.

**1.17 Transendothelial electrical resistance (TEER) assay**

In brief, 5×10^5^ HUVECs treated with or without DPDH were seeded into fibronectin-precoated Transwell inserts (0.4 μm pore polyester membrane) and incubated at 37°C overnight to form confluent monolayers. TEER values were measured using a WPI EVOM2 according to the manufacturer's instructions.

**1.18 Permeability assay**

Briefly, 5×10^5^ HUVECs treated with or without DPDH were seeded into fibronectin-precoated Transwell inserts (0.4 μm pore) and incubated at 37°C overnight to form confluent monolayers. FITC-dextran (40 kDa) was added to the upper chamber and incubated at 37°C for 60 min. Medium from the bottom chamber was collected into a 96-well black plate, and fluorescence was measured using a PerkinElmer Victor V plate reader (Ex/Em: 480/520 nm).

**Figure S1.** **CCM lesions at different time points.** a. Visual detection of CCM lesions in the hindbrains of P7 *Krit1^iECKO^* mice, P11 *Krit1^iECKO^* mice and P19 *Krit1^iECKO^* mice. Scale bar: 2 mm. b. Top panel: Representative immunofluorescence images of CD31 in the brain sections from P7 *Krit1^iECKO^*, P11 *Krit1^iECKO^* and P19 *Krit1^iECKO^* mice. Scale bar: 100 μm. The regions highlighted in the white dotted boxes are magnified at the bottom panel. Scale bar: 50 μm.

DPFD

**Figure S2. ^1^H NMR spectrum (500 MHz, CDCl_3_) of 1,6-dimethylphenanthro[1,2-b]furan-10,11-dione (DPFD).** ^1^H-NMR (500 MHz, CDCl_3_): δ (ppm) 9.24 (d, *J*=8.5 Hz, 1H), 8.30 (d, *J*=8.5 Hz, 1H), 7.81 (d, *J*=8.5 Hz, 1H), 7.55 (m, 1H), 7.35 (d, *J*=7 Hz, 1H), 7.30 (d, *J*=1.5 Hz, 1H), 2.69 (s, 3H), 2.29 (d, *J*=1 Hz, 3H).

 **Figure S3. ^13^C-NMR spectrum (125 MHz, CDCl_3_) of 1,6-dimethylphenanthro[1,2-b]furan-10,11-dione (DPFD).** ^13^C-NMR (125 MHz, CDCl_3_): δ (ppm) 183.60, 175.75, 161.25, 142.07, 135.21, 133.71, 132.97, 132.82, 130.70, 129.70, 128.40, 124.82, 123.26, 121.82, 120.54, 118.76, 19.88, 8.82.

DPFD

 **Figure S4. ^1^H NMR spectrum (500 MHz, CDCl_3_) of (R)-4,9-dimethyl-8,9-dihydrofuro[3,2-α]naphtho[2,1-c]phenazine (DDNP).** ^1^H-NMR (500 MHz, CDCl_3_): δ (ppm) 11.18 (d, *J*=8.5 Hz, 1H), 8.45 (d, *J*=8 Hz, 1H), 8.39 (d, *J*=9 Hz, 1H), 8.21 (m, 2H), 7.80 (m, 3H), 7.54 (d, *J*=6.5 Hz, 1H), 5.11 (t, *J*=9 Hz, 1H), 4.61 (m, 1H), 4.35 (m, 1H), 2.85 (s, 3H), 1.73 (d, *J*=7 Hz, 3H).

DDNP

 **Figure S5. ^13^C-NMR spectrum (125 MHz, CDCl_3_) of (R)-4,9-dimethyl-8,9-dihydrofuro[3,2-α]naphtho[2,1-c]phenazine (DDNP).** ^13^C-NMR (125 MHz, CDCl_3_): δ (ppm) 158.67, 143.34, 142.43, 141.01, 139.28, 134.62, 132.98, 132.32, 130.00, 129.96, 128.52, 128.08, 127.97, 127.70, 127.66, 127.10, 126.49, 124.24, 119.64, 119.01, 80.39, 37.09, 20.60, 20.05.

DDNP

DPDH

**Figure S6. ^1^H NMR spectrum (500 MHz, CDCl_3_) of (R)-1,6-dimethyl-11-phenyl-1,10-dihydro-2H-furo[2',3':1,2]phenanthro[3,4-d]imidazole (DPDH).** ^1^H NMR (500 MHz, CDCl_3_): δ (ppm) 10.54 (s, 1H), 8.21 (d, *J*=7.5 Hz, 2H), 8.10 (d, *J*=9 Hz, 1H), 8.02 (d, *J*=9 Hz, 1H), 7.72 (t, *J*= 8 Hz, 1H), 7.57 (t, *J*= 7.5 Hz, 2H), 7.50 (m, 2H), 5.00 (t, *J*= 8 Hz, 1H), 4.48 (t, *J*= 6.5 Hz, 1H), 4.08 (m, 1H), 2.84 (s, 3H), 1.60 (d, *J*=6.5 Hz, 3H).

 **Figure S7. ^13^C-NMR spectrum (125 MHz, CDCl_3_) of (R)-1,6-dimethyl-11-phenyl-1,10-dihydro-2H-furo[2',3':1,2]phenanthro[3,4-d]imidazole (DPDH).** ^13^C-NMR (125 MHz, CDCl_3_): δ (ppm) 153.62, 148.39, 145.51, 138.98, 134.12, 131.47, 130.44, 130.30, 129.42, 128.97, 127.42, 126.20, 126.18, 124.41, 123.51, 121.06, 120.45, 119.06, 115.82, 79.14, 36.65, 20.33, 19.54.

DPDH

**Figure S8. (+)-High‐resolution electrospray ionization‐mass spectrometry (HRESI-MS) spectra of DPFD, DDNP and DPDH.**

**Figure S9. DPDH serves as a potent MEKK3 inhibitor.** a. Co-IP analysis reveals that DPDH disrupts the MEKK3–MEK5 interaction. Data are representative of three biologically independent experiments. b. Virtual screening results of DPDH against 311 kinases from the KIPP database. c. The intersection of 311 potential kinase targets with 110 CCM disease-related kinases identified 46 overlapping kinases. d. Inhibition rates of 10 μM DPDH against 46 kinases.

**Figure S10.** **DPDH can be delivered to the lesion regions of CCMs and alleviate the CCM lesion burden.** a. Schematic diagram of experimental design. b. Visual appearance of CCM lesions in the hindbrains of *Ccm2^iECKO^* mice in the absence or presence of DPDH treatment, n=3-6. Scale bar: 2 mm. c. Representative LC-MS chromatogram of DPDH in the cerebra of *Krit1^iECKO^* mice. d. Pharmacokinetic parameters of DPDH in the cerebellum and cerebrum of *Krit1^iECKO^* mice. e. Representative LC-MS chromatogram of DHT in the cerebrum of *Krit1^iECKO^* mice. f. Representative LC-MS chromatogram of DHT in the cerebellum of *Krit1^iECKO^* mice. g. Quantification of the concentrations of DHT in the cerebellum and cerebrum of *Krit1^iECKO^* mice following a single injection of DHT (10 mg/kg, i.p.), n=3. h. Representative images of H&E staining for brain, heart, liver, spleen, lung, and kidney from different groups, n=3. Scale bar: 100 μm. All data are expressed as mean ± SD. ^**^*P* < 0.01, ns represents not significant.

**Figure S11.** **DPDH increases mural cell coverage of blood vessels and prevents vascular leakage in CCM lesions.** a. Quantification of VE-cadherin and ZO-1 coverage on CD31^+^ blood vessels in human CCM specimens, with three randomly selected fields analyzed per specimen. b. Representative images of whole-mount immunofluorescence staining for retinas isolated from the *Krit1^iECKO^* mice stained with CD31/a-SMA and CD31/NG2, n=3. Scale bars: 50 μm. c. Representative SEM images for the blood vessels in the hindbrains harvested from *Ccm2^iECKO^* mice in different groups. Scale bars: 10 μm. d. Representative immunofluorescence images of CD31 with fibrinogen alpha chain or CD41 in the brain sections from *Krit1^iECKO^* mice. The region highlighted in white dotted boxes are magnified on the right, n=3. Scale bar: 50 μm. e. Quantification of fibrinogen alpha chain-positive area (refer to d). f. Quantification of CD41-positive area (refer to d). g. Left: Representative flux image of cerebellar vasculature from *Krit1^ECKO^* mice via laser speckle contrast imaging. n=3. Bar, 2 mm. Right: Quantifications of the CBF by RFLSI software. The same region of interest (ROI) (white circle) was defined for all groups. n=3. LSCI measurements were performed using an RFLSI Analysis system with a color-coded velocity scale ranging from 0-80 mm/s: blue (0-20 mm/s, low flow), green (20-40 mm/s, moderate), yellow (40-60 mm/s, high), red (60-80 mm/s, maximum). System parameters included a frame rate of 25 Hz, a spatial resolution of 15.6 μm/pixel, a full cerebellum scanning region, and three regions per group for averaging. h. Representative visual inspection of brain tissues following the administration of EBD in the *Ccm2^iECKO^* mice in the absence or presence of DPDH treatment, n=3-5, Scale bar: 2 mm. i. Representative visual inspection of brain tissues following the administration of FITC-dextran in the *Ccm2^iECKO^* mice in the absence or presence of DPDH treatment, n=3. Scale bar: 2 mm. Quantification of leaked FITC-dextran by ImageJ software is shown. All data are expressed as mean ± SD. ^*^*P* < 0.05, ^**^*P* < 0.01, ^***^*P* < 0.001, ns represents not significant.

**Figure S12.** **DPDH fails to influence the mitogen‑activated protein kinase (MAPK) signaling cascades that are located at the downstream of MEKK3 in ECs.** b. The mRNA expression levels of *KRIT1* in the hCMEC/D3 cells transfected with si-*Control* or si-*KRIT1* were examined by qRT-PCR analysis. b. Representative immunofluorescence images for F-actin (green)/MEKK3 (red)/DPDH (blue) in the si-*KRIT1*-hCMEC/D3 cells. Scale bar: 100 μm. c. The mRNA expression levels of *KRIT1* in the hCMEC/D3 cells transfected with sh-*Control*-GFP or sh-*KRIT1*-GFP plasmids were examined by qPCR analysis (left), n=3. Representative images of hCMEC/D3 cells transfected with sh-*Control*-GFP or sh-*KRIT1*-GFP plasmids (right). d. The cell viability of sh-*KRIT1*-hCMEC/D3 cells was calculated *in vitro* by using the MTT assay after the treatment of different concentrations of DPDH, n=6. e. The mRNA expression levels of *KRIT1* in the HUVECs transfected with sh-*Control*-GFP and sh-*KRIT1*-GPF plasmids were examined by qPCR analysis (left), n=3. Representative images of HUVEC cells transfected with sh-*Control*-GFP or sh-*KRIT1*-GFP plasmids (right). f. Phosphorylated and total protein levels of P38, JNK, ERK1/2 in the different groups by western blot analysis. GAPDH was used as a loading control. Densitometric ratios for P38, JNK and ERK1/2 activities were quantified, n=3. g. Representative immunofluorescence images for pMLC2 (red)/DAPI (blue) in the hCMEC/D3 cells. Scale bar: 50 μm. h. Measurement of endothelial barrier function by TEER. i. The TEER values of HUVECs were measured by electrical impedance analysis, n=4. j. The permeability of HUVECs was quantified by measuring the fluorescence of FITC-dextran (40 kD) collected in the bottom chamber of transwell, n=6. All data are expressed as mean ± SD. ^*^*P* < 0.05, ^**^*P* < 0.01, ^***^*P* < 0.001.

**Figure S13. DPDH retards the microglia uptake of EC debris in the *Krit1*^iECKO^ mice.** a. The mRNA expression levels of *Ccl19* in the ECs isolated from *Krit1^iECKO^* mice in the absence or presence of DPDH treatment, n=5. b. Flow cytometry analysis of CD45^+^ cells isolated from *Krit1^iECKO^* mice in the absence of presence of DPDH treatment, n=3. c. Flow cytometry analysis of the percentage of CD4^+^ cells isolated from *Krit1^iECKO^* mice in the absence of presence of DPDH treatment, n=3. c. Flow cytometry analysis of the percentage of CD8^+^ cells isolated from *Krit1^iECKO^* mice in the absence of presence of DPDH treatment, n=3. e. Flow cytometry analysis of the percentage of microglial cells isolated from *Krit1^iECKO^* mice in the absence of presence of DPDH treatment, n=3. f. Representative immunofluorescence images of CD31 and IBA-1 in the brain sections from P7 and P11 *Krit1^iECKO^* mice, n=3. Scale bar: 50 μm. g. Representative immunofluorescence images of CD31 and IBA-1 in the brain sections from P14 and P19 *Krit1^iECKO^* mice, n=3. Scale bar: 50 μm. h. Schematic diagram of experimental design. i. Left: representative immunofluorescence images of CD31 and IBA-1 in the brain sections from P19 *Krit1^iECKO^* mice in the absence or presence of PLX5622 treatment, n=3. Scale bar: 100 μm. Right: Quantification for the number of microglial cells/mm^2^. j. Representative microCT images of CCM lesions in different groups. CCM lesions are shown in red, n=3. k. Representative immunofluorescence images of frozen sections stained with CD31 following the administration of FITC-dextran in the *Krit1^iECKO^* mice in the absence or presence of PLX5622 treatment, n=3. Scale bar: 100 μm. l. Quantification of microCT analysis in different groups (refer to j). m. Quantification of FITC-dextran-perfused area (refer to k). n. Quantification for the mRNA levels of *IL-6* and *IL-1β* in the HMC3 cells after the treatment of different concentrations of LPS, n=4. o. Quantification of the area of endothelial debris uptake by microglia in human CCM specimens, with three randomly selected fields analyzed per specimen. p. Quantification of IBA-1-positive area, n=3. q. Representative co-immunofluorescence staining for CD31, IBA-1 and CD68 in the cerebellum dissected from the *Krit1^ECKO^* mice, n=3. Scale bar: 20 μm. The white arrow indicated co-localization of IBA-1, CD31 and CD68. r. Flow cytometry analysis of the percentage of CD31^+^CX3CR1^+^ cells isolated from the *Krit1^iECKO^* mice in the absence of presence of DPDH treatment, n=3. s. Quantification for the co-expression of CD31 and CX3CR1 by flow cytometry analysis in different groups (refer to q). All data are expressed as mean ± SD. ^*^*P* < 0.05, ^**^*P* < 0.01, ^***^*P* < 0.001, ns represents not significant.

**Figure S14. DPDH attenuates microglial uptake of EC debris by inhibiting the MEKK3/NF-κB axis.** a. The mRNA expression levels of *MEKK3* in the HMC3 cells transfected with *sh-Control* or *sh-MEKK3* plasmids, n=3. b. Quantification for the expression of CD86 and CD206 by flow cytometry analysis in different groups. c. IL-6 and IL-1β levels in conditioned medium of each group, n=3. d. Representative immunofluorescence staining for MEKK3 and IBA-1 in the cerebella of different groups, n=3. Scale bar: 100 μm. The regions highlighted in white boxes are magnified. e. Representative immunofluorescence images for F-actin (green)/MEKK3 (red)/DPDH (blue) in the LPS-HMC3 cells, n=3. Scale bar: 100 μm. f. Phosphorylated and total protein levels of P38, JNK, ERK1/2 in the different groups by western blot analysis. g-i. Densitometric ratios for P38, JNK and ERK1/2 activities were quantified. GAPDH was used as a loading control. n=3. j. Left: Representative co-immunofluorescence staining for IBA-1 and IL-6 in the human CCM specimens, n=4. Scale bar: 50 μm. The regions highlighted in white dotted circles are magnified and the white arrows indicate co-localization of IBA-1 and IL-6. Right: Quantification of IL-6 area within IBA-1^+^ microglia in human CCM specimens. Three random fields were analyzed per specimen. k. Left: Representative co-immunofluorescence staining for IBA-1 and IL-1β in the human CCM specimens, n=4. Scale bar: 50 μm. The regions highlighted in white dotted circles are magnified and the white arrows indicate co-localization of IBA-1 and IL-1β. Right: Quantification of IL-1β area within IBA-1^+^ microglia in human CCM specimens. Three random fields were analyzed per specimen. All data are expressed as mean ± SD. ^*^*P*< 0.05, ^**^*P* < 0.01, ^***^*P* < 0.001, ns represents not significant.

**Figure S15.** **Gating strategy.** a. The gating strategy for Microglial cells.

**Figure S16. DPDH leads to additive effect on alleviating CCM lesion burden upon heterozygous endothelial depletion of MEKK3 in the *Ccm2^iECKO^Ccm2l^-/-^*** **mice.** a. Schematic diagram of experimental design. b. Visual inspection of brain CCM lesions in the *Ccm2^iECKO^*, *Ccm2^iECKO^Ccm2l^-/-^*, *Ccm2^iECKO^Ccm2l^-/-^Mekk3^het^* mice as well as *Ccm2^iECKO^Ccm2l^-/-^Mekk3^het^* mice treated with DPDH*.* Scale bar: 2 mm. c. MicroCT images of CCM lesions in the *Ccm2^iECKO^*, *Ccm2^iECKO^Ccm2l^-/-^*, *Ccm2^iECKO^Ccm2l^-/-^Mekk3^het^* mice as well as *Ccm2^iECKO^Ccm2l^-/-^Mekk3^het^* mice treated with DPDH. CCM lesions are shown in red, n=3-5. d. Quantification of microCT analysis in different groups. e. Representative whole-mount immunofluorescence images of IB4 for retinas isolated from the *Ccm2^iECKO^*, *Ccm2^iECKO^Ccm2l^-/-^*, *Ccm2^iECKO^Ccm2l^-/-^Mekk3^het^* mice as well as *Ccm2^iECKO^Ccm2l^-/-^Mekk3^het^* mice treated with DPDH, n=3. Scale bar: 500 μm. f. Quantification for the percentage of the marked condensed vascular plexus at the leading edge against the total area of the retinal blood vessels, n=3. g. Schematic diagram of experimental design. h. Top panel: visual appearance of CCM lesions in the hindbrains of mice in different groups, n=3. Scale bar: 2 mm. Bottom panel: Representative whole-mount immunofluorescence images of IB4 for retinas in different groups, n=3. Scale bar: 500 μm. i. Quantification for the percentage of the marked condensed vascular plexus at the leading edge against the total area of the retinal blood vessels, n=3. j. MicroCT imaging of CCM lesions in different groups. k. Quantification of microCT analysis in different groups, n=3. All data are expressed as mean ± SD. ^*^*P*< 0.05, ^**^*P* < 0.01, ^***^*P* < 0.001.

**Figure S17. DPDH attenuates fibrinogen extravasation and suppresses microglial aggregation within CCM lesions** a. The migration of HMC3 cells in different groups was determined by transwell co-culture system. n=3-5, Scale bar: 100 μm. Quantification for the number of transmigrated HMC3 cells is shown. b. Representative immunofluorescence images of frozen sections stained with CD31 following the administration of Fibrinogen-594 from *Krit1^iECKO^* mice, n=3. Scale bar: 100 μm. The regions highlighted in white boxes are magnified and the white arrows indicate that fibrinogen leaking from blood vessels. c. Quantification for the percentage of IBA-1, VWF and ICAM-1 positive area in the brain sections from *Krit1^iECKO^* mice, n=3. d. The mRNA expression levels of *Icam1* and *Vcam1* in the ECs isolated from the *Krit1^iECKO^* mice in the absence or presence of DPDH treatment. n=5. All data are expressed as mean ± SD. ^**^*P* < 0.01, ^***^*P* < 0.001, ns represents not significant.

**Supplementary Table S1. Primer sequences for qRT-PCR used in this study**

| Primer | Sequence |
| --- | --- |
| *GAPDH* (human) | Forward CAGGAGGCATTGCTGATGAT |
|  | Reverse GAAGGCTGGGGCTCATTT |
| *MEKK3* (human) | Forward AGATGTGGAGCACAAGGTGAC |
|  | Reverse ATTTATATCCCCTGCGGACTG |
| *KRIT1* (human) | Forward AATGGCAGAGAAGCATGAGCAGTG |
|  | Reverse ATTTCAGCATGTCCTCCTCCAGCA |
| *IL-6* (human) | Forward ACTCACCTCTTCAGAACGAATTG |
|  | Reverse CCATCTTTGGAAGGTTCAGGTTG |
| *IL-1β* (human) | Forward ATGATGGCTTATTACAGTGGCAA |
|  | Reverse GTCGGAGATTCGTAGCTGGA |
| *Gapdh* (mouse) | Forward AGGTCGGTGTGAACGGATTTG |
|  | Reverse TGTAGACCATGTAGTTGAGGTCA |
| *Ccl19* (mouse) | Forward GGGGTGCTAATGATGCGGAA |
|  | Reverse CCTTAGTGTGGTGAACACAACA |
| *Ccl11* (mouse) | Forward GAATCACCAACAACAGATGCAC |
|  | Reverse ATCCTGGACCCACTTCTTCTT |
| *Adamts4* (mouse) | Forward ATGGCCTCAATCCATCCCAG |
|  | Reverse AAGCAGGGTTGGAATCTTTGC |
| *Vcam1* (mouse) | Forward TTGACATCTCCCCCGGATCT |
|  | Reverse AACACAAGCGTGGATTTGGC |
| *Icam1* (mouse) | Forward AGCTCGGAGGATCACAAACG |
|  | Reverse AGGCCTGGCATTTCAGAGTC |
