# Supplementary material for: Inhibition of MEKK3 Prevents Cerebral Cavernous Malformation Progression Via Restoring Endothelial Integrity and Restricting Microglial Activation
Source: Adv Sci (Weinh). 2026 Oct 6:e78132. Online ahead of print. doi: 10.1002/advs.78132 (PMC13643112; doi:10.1002/advs.78132)
Supplement: Supplementary file 2 [file ADVS-9999-e78132-s005.docx]

**Western blot raw data in this study**
